# Serine/threonine/tyrosine kinase 1 drives pancreatic carcinogenesis via GSK3β sequestration-mediated Wnt/β-catenin pathway hyperactivation

**DOI:** 10.1038/s41392-025-02292-x

**Published:** 2025-06-30

**Authors:** Cefan Zhou, Xueying Dong, Shi Li, Yue Xi, Yuan Liu, Xuehong Qian, Ziyan Song, Li Zhou, Rui Zhang, Hao Lyu, Shuai Xiao, Dong Guo, Qi Zhang, Weiyong Liu, Yan Xiong, Zhentian Wang, Chaojun Yan, Zijian Zhang, Haichuan Zhu, Xing-Zhen Chen, Zhiyin Song, Jingfeng Tang

**Affiliations:** 1 National “111” Center for Cellular Regulation and Molecular Pharmaceutics, Key Laboratory of Fermentation Engineering (Ministry of Education), Hubei University of Technology, Wuhan, China; 2 Membrane Protein Disease Research Group, Department of Physiology, Faculty of Medicine and Dentistry, University of Alberta, Edmonton, AB Canada; 3 Animal Biosafety Level III Laboratory at the Center for Animal Experiment, Wuhan University, Wuhan, China; 4 Department of Clinical Laboratory, Tongji Hospital, Huazhong University of Science and Technology, Wuhan, China; 5 Zhongnan Hospital of Wuhan University, Institute of Hepatobiliary Diseases of Wuhan University, Transplant Center of Wuhan University, National Quality Control Center for Donated Organ Procurement, Hubei Key Laboratory of Medical Technology on Transplantation, Hubei Clinical Research Center for Natural Polymer Biological Liver, Hubei Engineering Center of Natural Polymer-based Medical Materials, Wuhan, China; 6 Department of Systems Biology for Medicine, School of Basic Medical Sciences, Fudan University, and Shanghai Fifth People’s Hospital, Fudan University, Shanghai, China; 7 Department of Pathology, School of Basic Medicine, Tongji Medical College and State Key Laboratory for Diagnosis and Treatment of Severe Zoonotic Infectious Diseases, Huazhong University of Science and Technology, Wuhan, China

**Keywords:** Cell biology, Biochemistry, Gastrointestinal cancer, Gastrointestinal cancer

## Abstract

The Wnt/β-catenin pathway is strongly relevant to pancreatic cancer progression, poor prognostic outcomes, and elevated cancer-related mortality. However, the mechanism underlying continuously activated Wnt/β-catenin signaling in pancreatic cancer, a context in which adenomatous polyposis coli (APC) mutations are rarely observed, remains poorly understood. In this study, we investigated the role of STYK1 in regulating canonical Wnt/β-catenin signaling and pancreatic cancer tumorigenesis using the *LSL-Kras*^*G12D*^; *Trp53*^*R172H/+*^; *Pdx1*^*Cre*^ mouse model. Our findings demonstrate that STYK1 directly binds to β-catenin and GSK3β, inhibiting GSK3β activity by increasing the level of its kinase-inactive form, which is phosphorylated at S9, and promoting its sequestration into MVBs. We further showed that STYK1-mediated GSK3β sequestration is impaired by autophagy inhibitors or in ATG7 knockout cells, linking this process to autophagic regulation. Structural analysis identified conserved tyrosine-based (Y191QRL194) and dileucine-based (GDLL203-204) sorting motifs in STYK1, which facilitate clathrin/AP2-dependent internalization essential for GSK3β sequestration. The phosphorylation of STYK1 at Y191 by BLK kinase enhances its interaction with AP2, thereby accelerating GSK3β sequestration and subsequent Wnt/β-catenin pathway activation. Notably, inhibitory peptides targeting either the STYK1-β-catenin or the STYK1-GSK3β interface significantly suppressed pancreatic cancer development in vitro and in vivo, underscoring their therapeutic potential. Collectively, these results elucidate a novel STYK1-driven mechanism for Wnt/β-catenin activation in APC-independent pancreatic cancer and provide preclinical evidence for targeting STYK1-mediated signaling as a therapeutic strategy.

## Introduction

Pancreatic ductal adenocarcinoma (PDAC), accounting for ~95% of pancreatic malignancies, remains one of the most lethal solid tumors with a 5-year survival rate below 13%.^1^ This dismal prognosis stems from its aggressive biological behavior characterized by early systemic dissemination, profound chemoresistance, and lack of effective therapeutic targets.^2^ While complete surgical resection offers the only curative potential, fewer than 20% of patients present with resectable disease due to delayed diagnosis.^3^ These clinical challenges underscore the critical need to decipher molecular mechanisms driving PDAC pathogenesis and identify novel therapeutic vulnerabilities.

Wnt ligand expression and activation of the canonical Wnt/β-catenin pathway have been associated and required for PDAC development.^4,5^ The cytoplasmic pool of β-catenin is continuously phosphorylated for degradation and maintained at low levels by the destruction complex. This complex includes glycogen synthase kinase 3β (GSK3β), the scaffolding protein Axin1, casein kinase 1α (CK1α), adenomatous polyposis coli (APC), and other proteins. GSK3β-mediated phosphorylation primes β-catenin for recognition by the E3 ligase β-TrCP, initiating ubiquitin-dependent proteolysis. This quality-control mechanism maintains β-catenin below nuclear threshold concentrations by intercepting its cytoplasmic-to-nuclear trafficking.^6,7^ The activation of the canonical Wnt/β-catenin pathway in response to Wnt stimulation requires the inactivation of the β-catenin destruction complex. Post-secretory trafficking enables Wnt ligands to interact with plasma membrane-anchored Frizzled (Fzd) receptors and their auxiliary partner, low-density lipoprotein receptor-related protein 5/6 (LRP5/6), at the cell surface. This interaction leads to the clustering of multiple receptors and ligands into a complex known as the signalosome.^8^ The formation of the signalosome triggers signal initiation and finally mediates negative regulation of GSK3β. GSK3β activity is strictly modulated by multiple molecular mechanisms, including site-specific phosphorylation events that dictate its functional state. Autophosphorylation at the Tyr216 residue stabilizes its catalytically active conformation (p-GSK3β Y216), while Ser9 phosphorylation induces a kinase-inhibited form by obstructing substrate binding. Under physiological conditions, GSK3β signaling maintains precise equilibrium through these regulatory nodes, but pathological disruption of this balance frequently leads to abnormal substrate phosphorylation patterns implicated in various disease pathologies. Such dysregulation underscores the enzyme’s central role in cellular homeostasis and its association with diverse clinical disorders.^9^

The endolysosomal network functions as a dynamic membranous hub essential for preserving proteostatic balance within cells. This system coordinates lipid and protein metabolism by mediating signaling cascades, secretory pathways, material recycling, and catabolic clearance processes. Following selective retrieval of reusable biomaterials to the plasma membrane or Golgi apparatus, degradation-targeted cargo undergoes lysosomal routing through late endosomal compartments. During this maturation phase, cytoplasmic proteins become packaged within intraluminal vesicles that bud inward from the bounding membrane, ultimately generating multivesicular organelles known as multivesicular bodies (MVBs).^10^ In this context, emerging models propose that Wnt signal transduction initiation involves endosome maturation dynamics, where internalization of Wnt-Fzd complexes drives MVBs biogenesis. This process creates topological isolation of GSK3β within MVB-enriched compartments, effectively establishing spatial segregation between the kinase and cytosolic substrates like β-catenin.^11^ This spatial segregation of GSK3β effectively restrains its kinase function toward β-catenin, thereby promoting β-catenin proteostasis and subsequent nuclear trafficking, where it activates TCF/LEF-dependent transcriptional programs that drive oncogenic gene networks.^12^ While malignancies such as colorectal cancer frequently exhibit Wnt pathway-activating mutations in canonical regulators (e.g., APC and β-catenin), PDAC pathogenesis unfolds through non-canonical mechanisms devoid of these genetic alterations.^13^ Mechanisms underlying constitutive Wnt signaling in PDAC, especially that regulating GSK3β sequestration, remain to be identified.

Serine/threonine/tyrosine kinase 1 (STYK1), a proto-oncogenic transmembrane receptor alternatively termed NOK, which consists of a kinase domain, intracellular domain (ICD), and transmembrane domain, was identified as an oncogene with high transformation potential in multiple cancer types.^14–17^ Recently, our group recognized that STYK1 depletion disrupts autophagosome biogenesis, establishing it as a critical modulator of autophagic flux. Mechanistically, STYK1 scaffolds autophagic initiation by forming direct complexes with PtdIns3K-C1, thereby enhancing BECN1 phosphorylation at serine residues, a post-translational modification that attenuates BECN1-BCL2 binding affinity.^18^ This contrasts with established EGFR hyperactivation paradigms (mutant or ligand-stimulated wild-type), which induce BECN1 tyrosine phosphorylation to enforce autophagic suppression.^19^ Importantly, STYK1 antagonizes EGFR-driven phosphorylation cascades, preserving BECN1’s functional interactome required for autophagosome nucleation.^15^ STYK1 was also reported as an oncogenic amplifier that hijacks KRAS effector networks, specifically the PI3K/AKT and MEK/ERK signaling axes, to drive tumor progression across malignancies. This molecular paradigm gains particular relevance in PDAC, where somatic KRAS mutations dominate (~95% prevalence) and functionally orchestrate pancreatic carcinogenesis from its earliest premalignant stages, notably during pancreatic intraepithelial neoplasia (PanIN) precursor lesions. This prompted a focused investigation into STYK1’s functional implications within pancreatic cancer. By using multiple in vitro and in vivo assays, we figured out that higher STYK1 expression is related to poor pancreatic cancer survival and that STYK1 depletion suppresses pancreatic cancer cell development. We also highlight the role of STYK1 in accelerating Wnt/β-catenin signaling by binding with GSK3β and β-catenin. Moreover, we showed that clathrin/AP2-mediated internalization of STYK1 and sequestration of GSK3β played essential roles in the inactivation of the β-catenin destruction complex and subsequent Wnt/β-catenin signaling. Additionally, the effects of STYK1-derived peptides that target its interaction with GSK3β and β-catenin were also investigated in pancreatic cancer mice models.

## Results

### STYK1 deletion alleviates pancreatic cancer progression

**Fig. 1:**
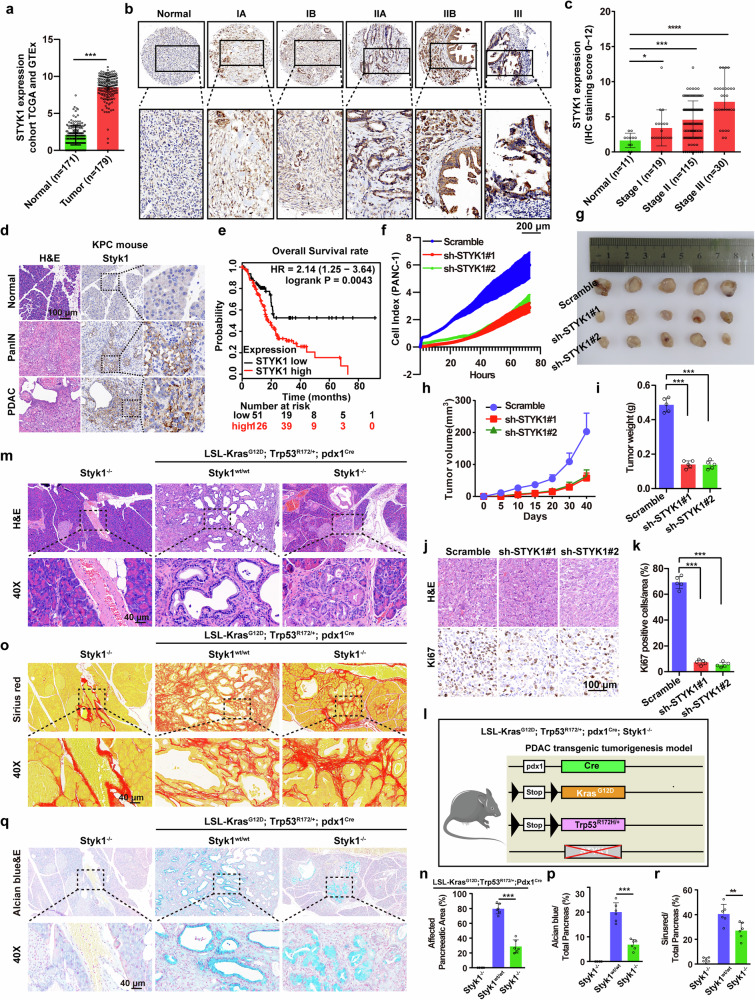
STYK1 is upregulated and STYK1 deletion alleviates pancreatic cancer progression. **a** Relative *STYK1* mRNA level in TCGA and GETx databases of pancreatic cancer patients’ tissues. **b**, **c** Comparative IHC analysis of STYK1 expression levels between pancreatic cancer and normal tissues, with quantitative scoring. Scale bar: 200 μm. **d** Styk1 IHC staining patterns across normal pancreas, PanIN, and PDAC tissues in mice. Scale bar: 100 μm. **e** STYK1 prognostic significance in pancreatic cancer via Kaplan-Meier survival analysis. **f** STYK1 knockdown altered PANC-1 cell growth kinetics in real-time impedance assays (16-well E-plate, *n* = 3). Tumor specimens from STYK1-depleted groups were photographed (**g**), with growth curves (**h**, *n* = 5) and final tumor weights (**i**, *n* = 5) quantified across experimental cohorts. **j**, **k** Representative H&E staining, immunohistochemical images, and the quantification of Ki67 in excised tumor tissues (*n* = 5). Scale bar: 100 μm. **l** The diagram depicts the strategy for the generation of KPC; Styk1 KO mice (KPCS). **m**, **n** Representative histological images and the quantification of H&E staining in the indicating groups. Scale bar: 40 μm. **o**–**r** Representative histological images and the quantification of sirius red or alcian blue staining in the indicating groups (*n* = 6). Scale bar: 40 μm. Data were represented as mean ± SD, *p < 0.05; **p < 0.01; ***p < 0.001

Interrogation of the GEPIA2 transcriptomic database revealed that *STYK1* is overexpressed in several types of human cancers, especially pancreatic cancer (Supplementary Fig. 1a). Multi-platform validation across GTEx, TCGA, and four independent GEO cohorts consistently obtained similar results (Fig. 1a, Supplementary Fig. 1b–e). Immunohistochemical (IHC) profiling of pancreatic cancer tissue microarrays revealed stage-dependent STYK1 overexpression, showing marked elevation in pancreatic cancer cells (Fig. 1b, c). We further tested STYK1 expression in tissues from the classical *LSL-Kras*^*G12D*^; *Trp53*^*R172H/+*^; *Pdx1*^*Cre*^ mice (KPC mice), which is a well-established model for PDAC development. Mirroring clinical observations, mouse PanIN lesions exhibited neoplastic upregulation of STYK1 compared to non-dysplastic counterparts, with expression gradients demonstrating stage-correlated intensification paralleling PDAC advancement (Fig. 1d). Multivariate regression of IHC data from pancreatic cancer tissue microarrays demonstrated STYK1 overexpression exhibits significant covariance with TNM stage, tumor size and perineural invasion, a pathognomonic feature of locally advanced or metastatic PDAC, serves as an independent prognostic indicator correlating with aggressive disease progression and reduced survival outcomes (Supplementary Fig. 1f).^20^ Moreover, survival modeling via log-rank test confirmed elevated STYK1 expression portends diminished median overall survival (OS) (Fig. 1e). These clinicopathological correlations position STYK1 as a pathognomonic driver of PDAC aggressiveness.

To investigate the role of STYK1 in pancreatic cancer proliferation, multiple in vitro and in vivo assays were performed. STYK1 depletion significantly reduced RTCA-measured cell index, reflecting reduced cancer cell proliferation capacity (Fig. 1f, Supplementary Fig. 1g). The ability of DNA synthesis was decreased in STYK1-depleted AsPC-1 and PANC-1 cells by the 5-ethynyl-20-deoxyuridine incorporation assay (Supplementary Fig. 1h, i). We next constructed tumor xenograft models by subcutaneously injecting PANC-1 cells to investigate the role of STYK1 in pancreatic cancer in vivo. We found that depletion of STYK1 in mouse xenograft models resulted in markedly attenuated tumor growth kinetics relative to control cohorts (Fig. 1g). Tumor burden assessments (volume and weight) revealed substantial reductions in STYK1-depleted groups compared to shRNA-control implants (Fig. 1h, i). IHC profiling further demonstrated suppression of proliferative indices, evidenced by decreased Ki67 immunoreactivity in STYK1-knockdown specimens (Fig. 1j, k). To directly investigate the role of STYK1 in regulating PDAC progression, we introduced *Styk1* knockout by CRISPR/Cas9-mediated depletion of the second, third, and fourth exons of *Styk1* open reading frame in KPC mice (Fig. 1l, Supplementary Fig. 1j, m), and generated *LSL-Kras*^*G12D/+*^; *Trp53*^*R172H/+*^; *Pdx1*^*Cre*^; *Styk1*^*−/−*^ mice (KPCS mice). As the KPC mice progressed to advanced PDAC within 3–6 months,^21^ we sacrificed and isolated pancreatic tissues from 12-week-old KPC and KPCS mice. Histological analysis revealed a predominant decrease in the area of pancreata that was replaced by PanIN or PDAC in KPCS mice compared with KPC mice, whereas no influence in *Styk1*^*−/−*^ mice (Fig. 1m, n). Moreover, we determined whether loss of Styk1 has any effect on the desmoplastic reaction and glycophenotype that are highly observed and essential for the oncogenic features of cell growth and invasion.^22,23^ We performed sirius red and alcian blue staining to visualize and quantify the collagen and mucin, respectively. Increased abundance of collagen and mucin were found in KPC mice and significantly decreased in KPCS mice (Fig. 1o–r). Taken together, these results indicated that loss of STYK1 inhibits pancreatic cancer proliferation and delays PDAC progression.

### STYK1 enhances pancreatic cancer tumorigenicity by promoting canonical Wnt/β-catenin signaling

**Fig. 2:**
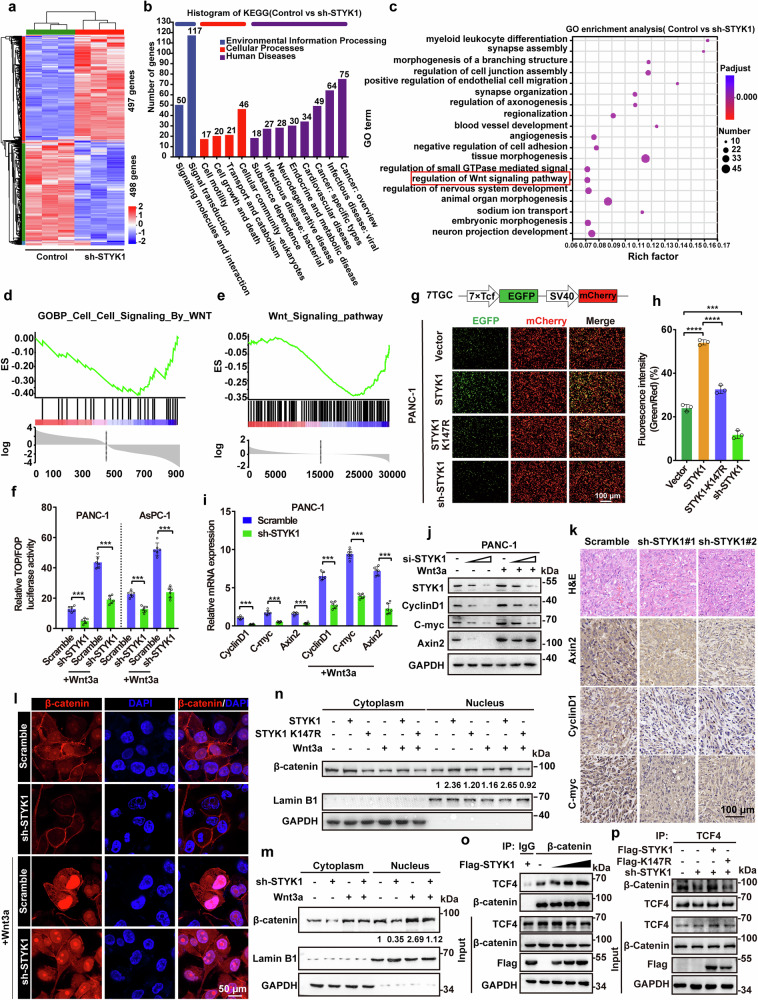
STYK1 promotes canonical Wnt/β-catenin signaling. **a** Heatmap of gene expression of PANC-1 cells after STYK1 depletion. **b**, **c** Kyoto Encyclopedia of Genes and Genomes (KEGG) and Gene Ontology (GO) pathway enrichment analysis of differentially expressed genes after STYK1 depletion. **d**, **e** GSEA analysis of enriched gene set in the Wnt/β-catenin signaling comparison of normal and STYK1-depleted PANC-1 cells and TCGA pancreatic cancer database with distinguishing STYK1 level. **f** TOP/FOPflash reporter assays quantified Wnt activity in PANC-1/AsPC-1 cells in response to Wnt3a treatment (100 ng/mL, 4 h; *n* = 6). **g**, **h** 7TGC plasmid schematic and fluorescence imaging of PANC-1 cells with STYK1 knockdown or WT/K147R mutant expression, with quantified GFP/RFP intensity ratios (*n* = 3). Scale bar: 100 μm. **i**, **j** mRNA (*n* = 6) and protein levels of Wnt targets (CyclinD1, C-myc, Axin2) in PANC-1 cells following STYK1 depletion with or without Wnt3a treatment. **k** Representative immunohistochemical images of Axin2, CyclinD1, and C-myc in excised tumors tissues. Scale bar: 100μm. **l** Confocal microscopy analysis of β-catenin localization in PANC-1 cells following STYK1 depletion with or without Wnt3a treatment. Scale bar: 50 μm. **m** Western blot analysis of nuclear β-catenin levels in STYK1-depleted PANC-1 cells with or without Wnt3a treatment. **n** Western blot analysis of nuclear β-catenin levels in HEK293T cells overexpressing WT or K147R mutant STYK1 with or without Wnt3a treatment. **o**, **p** Endogenous TCF4/β-catenin interaction in PANC-1 cells following STYK1 depletion or WT/K147R mutant overexpression. Data were represented as mean ± SD, *p < 0.05; **p < 0.01; ***p < 0.001

To gain insights into the molecular mechanisms underlying the role of STYK1 in pancreatic cancer tumorigenicity, we performed global gene expression analysis of pancreatic cancer cells that were stably transfected with STYK1 shRNA by RNA sequencing (RNA-Seq). The results showed that 498 genes were down-regulated and 497 genes were up-regulated in PANC-1 cells that harbored STYK1 depletion (*p* < 0.05) (Fig. 2a). The differentially expressed genes were mainly enriched in signaling molecules and interaction, signal transduction, cellular community, and as expected, in cancer development (Fig. 2b). Gene ontology enrichment analysis showed that STYK1 depletion altered the regulation of the Wnt/β-catenin signaling pathway, that thought to be strongly relevant to pancreatic cancer progression (Fig. 2c).^24,25^ Moreover, gene set enrichment analysis (GSEA) indicated that the set of genes that were down-regulated in STYK1-depleted PANC-1 cells, or the pancreatic cancer patients expressing low levels of STYK1 from the TCGA database, showed enrichment for gene sets associated with Wnt signaling, Wnt activated receptor activity and Wnt related protein binding (Fig. 2d, e, Supplementary Fig. 2a, b).

Given the centrality of β-catenin-dependent transcriptional machinery in pancreatic oncogenesis, we interrogated STYK1’s regulatory crosstalk with canonical Wnt signaling. Dual luciferase reporter systems (TOPflash/FOPflash) in AsPC-1 and PANC-1 PDAC models revealed STYK1 ablation suppresses both basal and Wnt3a-stimulated β-catenin transactivation (Fig. 2f). Complementary validation using a fluorescence-based TCF/LEF transcriptional biosensor (7TGC system) confirmed STYK1’s modulatory effects on β-catenin-driven transcriptional flux (Fig. 2g, h). In contrast, STYK1 overexpression increased the transactivating activity of β-catenin with or without iCRT-14 (a Wnt inhibitor that inhibits TCF-β-catenin interaction) treatment, whereas the STYK1 kinase-dead mutant that was generated by replacing the ATP binding lysine reside with arginine (K147R)^18,26^ showed no effects compared with wild-type STYK1, suggesting the kinase activity of STYK1 involved in regulating Wnt/β-catenin signaling (Fig. 2g, h, Supplementary Fig. 2c). We further investigated the level of Wnt/β-catenin target genes after STYK1 depletion. Consistent with the RNA-seq data, STYK1 depletion or its kinase-dead mutant significantly reduced both the mRNA and protein levels of CyclinD1, C-myc, and Axin2 in PANC-1 and AsPC-1 cells compared with normal or wild-type STYK1 transfected cells, respectively (Fig. 2i, j, Supplementary Fig. 2d–h). STYK1 depletion-induced down-regulation of Wnt/β-catenin target genes was further confirmed by IHC staining of the xenograft tumor tissues (Fig. 2k). Furthermore, STYK1 silencing attenuated β-catenin nuclear translocation under both basal and Wnt3a-stimulated conditions (Fig. 2l, m). Conversely, STYK1 overexpression amplified β-catenin nuclear accrual with or without Wnt3a induction, whereas its kinase-dead mutant showed alleviated effects (Fig. 2n). To delineate the TCF4 recruitment mechanism, co-immunoprecipitation assays demonstrated STYK1 enhances β-catenin-TCF4 complex assembly in a kinase activity-dependent manner (Fig. 2o, p). Collectively, these findings position STYK1 as a kinase-driven modulator of canonical Wnt signaling efficacy.

### STYK1 binds GSK3β and β-catenin and inhibits cytoplasmic GSK3β activity thus stabilizing β-catenin

**Fig. 3:**
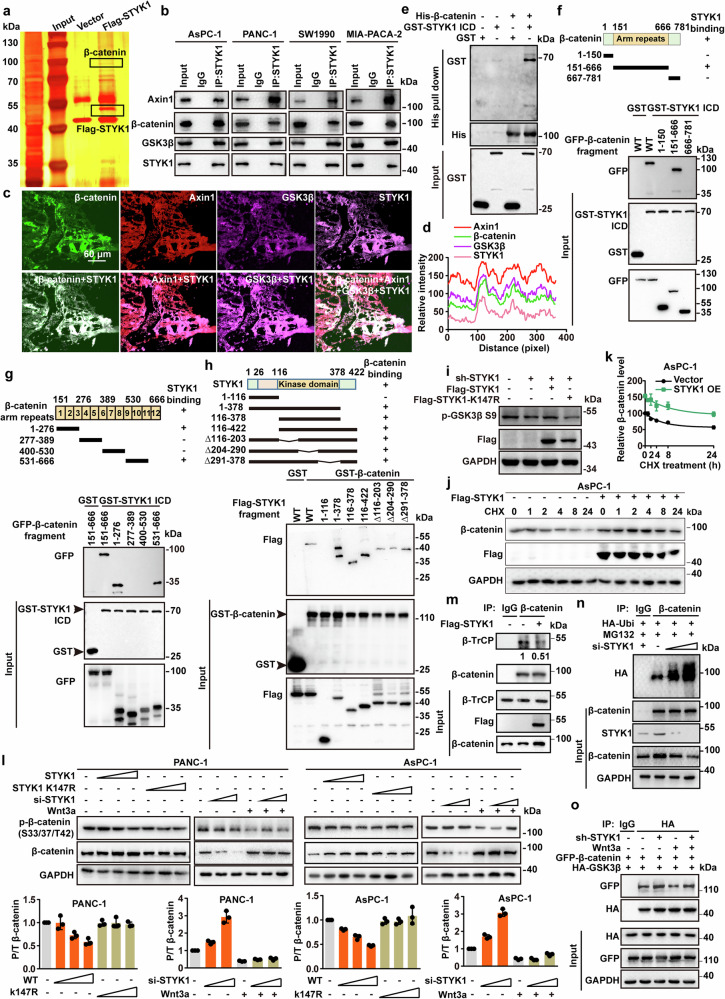
STYK1 binds GSK3β and β-catenin and inhibits cytoplasmic GSK3β activity thus stabilizing β-catenin. **a** Flag-tagged STYK1 immunoprecipitates from transfected HEK293T cells analyzed by silver staining. **b** Endogenous STYK1-GSK3β-β-catenin interactions were examined by co-IP/Western across pancreatic cancer cell lines (AsPC-1, PANC-1, SW1990, MIA-PACA-2). **c**, **d** Immunofluorescent staining of endogenous STYK1, Axin1, GSK3β, and β-catenin in pancreatic cancer clinical tissues. Plot profile analysis of protein colocalization. The X-axis represents the distance in pixels along the region of interest, and the Y-axis indicates the relative fluorescence intensity of each channel. **e** GST-STYK1 and His-β-catenin were co-incubated, pulled down with Ni-sepharose, and analyzed by Western blot. **f**–**h** GST pull-down assays analyzed interactions between STYK1 ICD/β-catenin and their truncation mutants (diagrammed) in HEK293T transfectants. **i** Western blot analysis of p-GSK3β S9 after wild-type STYK1 and its K147R mutant transfection. **j**, **k** Western blot analysis of β-catenin after STYK1 overexpression in the CHX chase assay (*n* = 3). **l** Western blot analysis quantified active-β-catenin and p-β-catenin (S33/S37/T41) levels following STYK1 knockdown/overexpression upon Wnt3a treatment (*n* = 3). **m** STYK1 overexpression disrupted endogenous β-TrCP/β-catenin interactions in PANC-1 cells, as demonstrated by co-IP/Western blot analysis. **n** STYK1 knockdown enhanced β-catenin ubiquitination in a dose-dependent manner in MG132-treated AsPC-1 cells, as confirmed by Western blot analysis. **o** Co-IP/Western blot analysis of HA-GSK3β/GFP-β-catenin interactions in STYK1-depleted PANC-1 cells with or without Wnt3a treatment. Data were represented as mean ± SD, *p < 0.05; **p < 0.01; ***p < 0.001

To further investigate the mechanisms of STYK1 in the regulation of Wnt/β-catenin signaling, we performed Flag-affinity purification of STYK1-associated protein complexes from HEK293T cells. Subsequent LC-MS/MS analysis identified GSK3β and β-catenin as prominent interactors, evidenced by multiple unique peptide spectra matching these proteins (Fig. 3a), suggesting that SYTK1 functions in Wnt/β-catenin signaling through regulating the activity of β-catenin destruction complex. Expectedly, the endogenous interaction between GSK3β, β-catenin, and STYK1 was confirmed using immunoprecipitation and immunofluorescence assays with specific antibodies in AsPC-1, PANC-1, SW1990, and MIA-PACA-2 cells and pancreatic cancer patient's tissues (Fig. 3b–d, Supplementary Fig. 3a, b). Moreover, STYK1 also successfully pulldown Axin1, another core scaffold protein of the β-catenin destruction complex (Fig. 3b–d, Supplementary Fig. 3a, b). To validate these associations, we employed recombinant GST pull-down assays in the bacterial expression system. However, we have not obtained the purified full-length STYK1, perhaps owing to the hydrophobic surfaces, flexibility, and instability of membrane proteins. Alternatively, we generated GST-tagged STYK1 ICD by truncating its ectodomain and transmembrane regions, and His-tagged β-catenin and GSK3β. The results revealed that purified GST-STYK1 bound directly to His-GSK3β and His-β-catenin (Fig. 3e, Supplementary Fig. 3c). We also mapped the binding domain of STYK1 with β-catenin and GSK3β, and we found that STYK1 could bind the Arm repeats region (151-666 aa) of β-catenin and the kinase domain (123-353 aa) of GSK3β (Fig. 3f, Supplementary Fig. 3d). Given the essential role of the arm repeats of β-catenin in binding with multiple regulatory factors,^27^ we further recognized that the Arm repeats 1-3 (151-276 aa) and 10-12 (530-666 aa) of β-catenin mediated its interaction with STYK1 from the results of the co-immunoprecipitation assays (Fig. 3g). Additionally, we found that STYK1 interacted with both GSK3β and β-catenin through its kinase domain (Fig. 3h, Supplementary Fig. 3e).

As the Arm repeats 2-4 of β-catenin were reported essential for its interaction with Axin1,^28^ where the Arm repeats 2 and 3 overlapped with the binding region with STYK1, we, therefore, asked whether STYK1 has functions in the activity of GSK3β and the stability of β-catenin. The results revealed that STYK1 knockdown decreased the level of p-GSK3β S9 (indicating enhanced kinase activity), while wild-type but not kinase-dead inactive STYK1 restored p-GSK3β S9 levels, consistent with prior findings (Fig. 3i).^9,29^ Cycloheximide (CHX) chase assay revealed that STYK1 overexpression significantly inhibited the degradation of β-catenin, resulting in increased β-catenin level. In contrast, STYK1 knockdown accelerated the downregulation of β-catenin protein levels in PANC-1 and AsPC-1 cells (Fig. 3j, k, Supplementary Fig. 3f–k). To examine the effect of STYK1 knockdown on the competency of the β-catenin destruction complex, levels of p-β-catenin (S33/37/T41) and total β-catenin were measured in PANC-1 and AsPC-1 cells. The results showed that STYK1 knockdown increased the ratio of phosphorylated β-catenin to total β-catenin, whereas wild-type STYK1, not its kinase-dead mutant decreased the ratio of phosphorylated β-catenin to total β-catenin (Fig. 3l). Moreover, the results also showed that STYK1 expression reduced the recruitment of β-TrCP to β-catenin, and STYK1 knockdown increased the level of ubiquitinated β-catenin, respectively (Fig. 3m, n, Supplementary Fig. 3l). Additionally, STYK1 knockdown enhanced GSK3β-β-catenin binding with or without Wnt3a stimulation, while wild-type (but not kinase-inactive) STYK1 overexpression dose-dependently disrupted β-catenin-Axin1 interactions (Fig. 3o, Supplementary Fig. 3m).

### STYK1 promotes cell membrane-associated GSK3β sequestration in an ESCRT-dependent manner

**Fig. 4:**
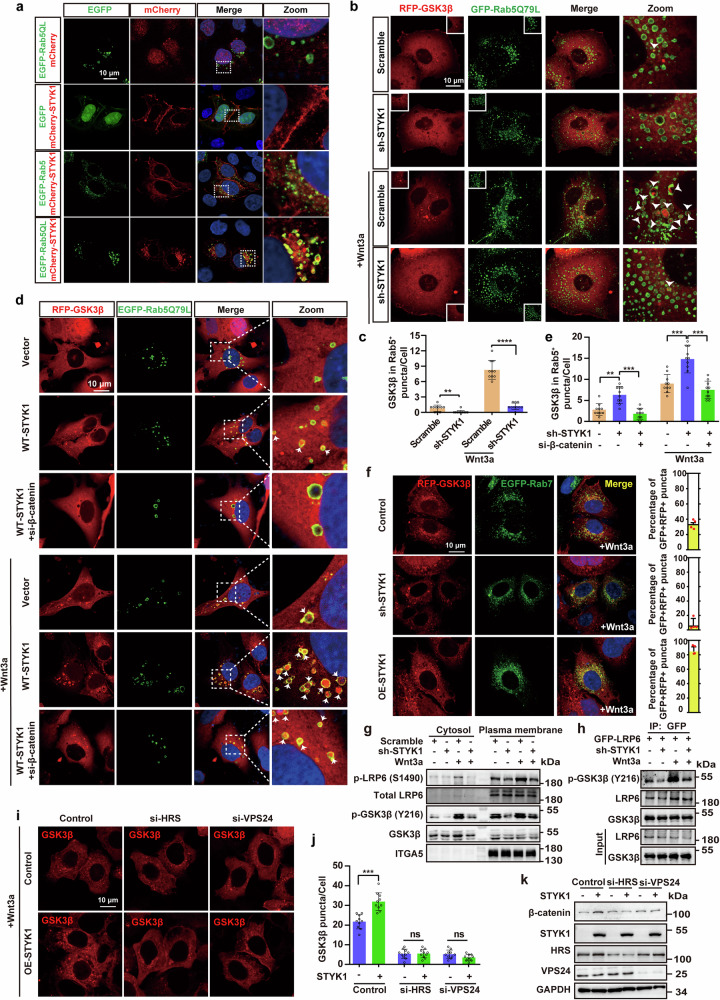
STYK1 promotes cell membrane-associated GSK3β sequestration in an ESCRT-dependent manner. **a** Confocal microscopy images of mCherry-tagged STYK1 and EGFP-tagged Rab5 and its Q79L mutant in HeLa cells. Scale bar: 10 μm. **b**, **c** Representative immunofluorescence images of RFP-tagged GSK3β and EGFP-tagged Rab5 Q79L mutant, and the quantification of the amount of GSK3β within Rab5^+^ endosomes in response to Wnt3a treatment after STYK1 depletion in PANC-1 cells (*n* = 10). Scale bar: 10 μm. **d**, **e** Representative immunofluorescence images of RFP-tagged GSK3β and EGFP-tagged Rab5 Q79L mutant, and the quantification of the amount of GSK3β within Rab5^+^ endosomes with or without Wnt3a treatment after STYK1 overexpression and β-catenin knockdown in human bone osteosarcoma epithelial (U2OS) cells (*n* = 10). Scale bar: 10 μm. **f** Representative immunofluorescence images and the quantification of the co-localization between Rab7 and RFP-tagged GSK3β upon Wnt3a treatment (*n* = 3). Scale bar: 10 μm. **g** Western blot analysis of p-LRP6 S1490, GSK3β, and the active form p-GSK3β Y216 levels after STYK1 depletion using plasma membrane separation assays in PANC-1 cells. **h** The level of p-GSK3β Y216 in the LRP6 immunoprecipitate by western blot. **i**, **j** GSK3β puncta formation in response to Wnt3a treatment was assessed by immunofluorescence imaging and quantified in PANC-1 cells with HRS/VPS24 depletion (*n* = 10). Scale bar: 10 μm. **k** The level of β-catenin after STYK1 overexpression in the PANC-1 cell lysates with HRS or VPS24 knockdown by western blot assays

As we previously reported STYK1 localized partly in the cell membrane^15^ and the intracellular parts co-localized with Rab5 or Rab7 positive (Rab5^+^ or Rab7^+^) endosomes (Fig. 4a, Supplementary Fig. 4a, b) that related with GSK3β sequestration, we further asked whether STYK1 affected GSK3β sequestration into multivesicular bodies. To this end, the constitutively active Rab5 Q79L mutant was engineered to induce enlarged late endosomal compartments.^30^ As expected, mCherry-tagged STYK1 was detected in Rab5 Q79L^+^ late endosomes (Fig. 4a). GSK3β subcellular distribution was then analyzed in Rab5 Q79L-expressing cells. The results revealed that stably expressed GSK3β was diffuse throughout the cell but fewer within the Rab5^+^ endosomes (Fig. 4b). Wnt3a remarkably increased the amount of GSK3β within Rab5^+^ endosomes, which were reduced upon STYK1 depletion (Fig. 4b, c). In contrast, STYK1 expression increased the amount of GSK3β within Rab5^+^ endosomes in response to Wnt3a treatment (Fig. 4d, e). β-catenin was reported to be required for GSK3β sequestration,^11^ considering our data that STYK1 also binds β-catenin, we tested whether β-catenin affected STYK1-mediated GSK3β sequestration. The results revealed that β-catenin knockdown indeed reduced the amount of GSK3β within Rab5^+^ endosomes after STYK1 overexpression (Fig. 4d, e). Moreover, STYK1 deficiency showed significantly reduced lysobisphosphatidic acid (LBPA) puncta, another late-endosomal marker compared with control cells, whereas no alteration of the early-endosomal marker EEA1 (Supplementary Fig. 4c, d). STYK1 depletion also significantly decreased endogenous GSK3β punctate structures (Supplementary Fig. 4e). In addition, GSK3β showed detectable co-localization with Rab7 and CD63, another late endosome or MVB marker, which suggests the presence of GSK3β in MVBs under Wnt3a treatment (Fig. 4f, Supplementary Fig. 4f, g). The colocalization was significantly decreased, whereas increased after STYK1 depletion or overexpression, respectively (Fig. 4f, Supplementary Fig. 4f, g). Wnt3a-induced sequestration is mainly triggered by the phosphorylation at LRP6 PPPS(1490)P motif by GSK3β, which subsequently amplificated and formed LRP6 signalosome.^31,32^ We also found that STYK1 depletion remarkedly decreased the signal of phosphorylated LRP6 S1490 in cell membrane fraction upon Wnt3a treatment (Fig. 4g). Plasma membrane-associated pools of GSK3β were reported essential for LRP6 phosphorylation and signalosome formation,^32^ we also showed that STYK1 depletion significantly reduced the level of p-GSK3β Y216, which is representative of a kinase-active form, but no alteration of total GSK3β level in the plasma membrane fraction (Fig. 4g). Moreover, we also found that LRP6-bound phosphorylated GSK3β (p-GSK3β Y216) was significantly reduced upon STYK1 depletion with or without Wnt3a treatment (Fig. 4h), indicating STYK1 is essential for LRP6 phosphorylation and signalosome formation. ESCRT machinery was reported involving Wnt-induced GSK3β sequestration into MVBs,^11^ we then analyzed the role of ESCRT machinery on STYK1-mediated GSK3β sequestration. ESCRT-0 (HRS) and ESCRT-III (VPS24) depletion impaired STYK1-mediated GSK3β/LBPA vesicular sequestration and β-catenin accumulation, revealing ESCRT-dependent regulation of Wnt signaling components (Fig. 4i-k, Supplementary Fig. 4h, i).

### Disrupting STYK1-β-catenin or STYK1-GSK3β interaction inhibits GSK3β sequestration and subsequent Wnt/β-catenin signaling

**Fig. 5:**
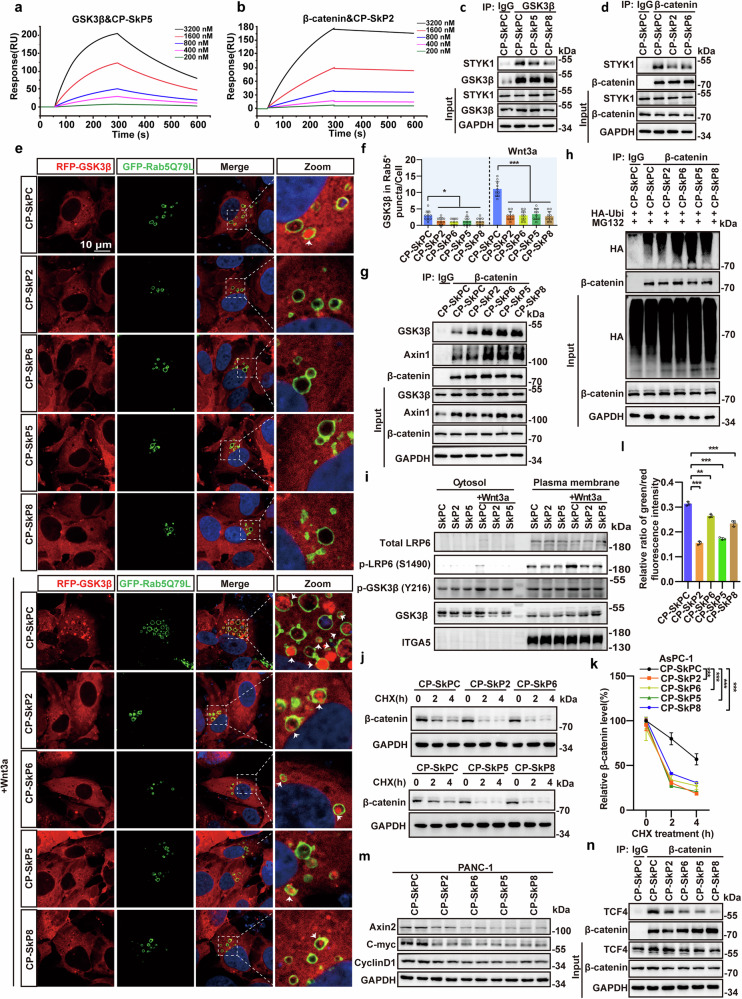
Disrupting STYK1-β-catenin or STYK1-GSK3β interaction inhibits GSK3β sequestration and subsequent Wnt/β-catenin signaling. **a**, **b** SPR analysis quantified binding kinetics between TAT-conjugated peptides and β-catenin/GSK3β. **c**, **d** Western blot analysis of the binding of endogenous GSK3β and β-catenin with STYK1 after chemically synthesized STYK1-driving peptides treatment using indicated antibodies. **e**, **f** Representative immunofluorescence images of RFP-tagged GSK3β and EGFP-tagged Rab5 Q79L mutant, and the quantification of the amount of GSK3β within Rab5^+^ endosomes with or without Wnt3a treatment after STYK1-driving peptides treatment in U2OS cells (*n* = 10). Scale bar: 10 μm. **g** Endogenous interaction between Axin1, GSK3β and β-catenin in AsPC-1 cells upon STYK1-driving peptides treatment was analyzed by western blot. **h** Western blot analysis of the level of β-catenin ubiquitination in PANC-1 cell lysates after STYK1-driving peptides treatment. **i** Western blot analysis of the level of cytoplasmic or membranal p-LRP6 S1490 and p-GSK3β Y216 level in PANC-1 cells with or without Wnt3a treatment. ITGA5 was used as a cell plasma membrane positive control. **j**, **k** Western blot analysis of the level of β-catenin after STYK1-driving peptides treatment in the CHX chase assay in AsPC-1 cells (*n* = 3). **l** Fluorescence microscopy was performed to assess STYK1-driving peptides in PANC-1 cells, with the green/red fluorescence intensity ratio quantified for analysis (*n* = 3). **m** Wnt target proteins (CyclinD1, C-myc, Axin2) in PANC-1 cells treated with STYK1-driving peptides. **n** Endogenous TCF4/β-catenin interactions in PANC-1 cells following STYK1-driving peptides treatment. Data were represented as mean ± SD, *p < 0.05; **p < 0.01; ***p < 0.001

To investigate the role of STYK1-β-catenin or STYK1-GSK3β interaction in GSK3β sequestration and subsequent Wnt/β-catenin signaling, we generated peptides that specifically disrupted the binding of STYK1 to either β-catenin or GSK3β. To this end, nine α-helices derived from the STYK1 protein kinase domain mediated the interaction with both β-catenin and GSK3β using the predictive I-TASSER server (Supplementary Fig. 5a).^33^ Screening of nine GFP-conjugated α-helical STYK1 peptides ((GGGGS)n linkers) identified the fifth and eighth STYK1-driven peptides (hereafter referred to as SkP) SkP5/SkP8 as critical mediators of STYK1-GSK3β binding and Wnt target gene activation, outperforming SkP1 and the nonbinding N-terminal control peptide (SkPC) (Supplementary Fig. 5b). Moreover, SkP2 and SkP6 showed the most binding ability to β-catenin (Supplementary Fig. 5c). We then chemically synthesized SkP5, SkP8, SkP2, and SkP6 linking with a cell-penetrating peptide TAT, which was named CP-SkP5, CP-SkP8, CP-SkP2 and CP-SkP6, respectively. By using the surface plasmon resonance (SPR) analysis, we found that CP-SkP5 and CP-SkP8 showed direct binding with GSK3β, whereas CP-SkP2, CP-SkP6 directly bound with β-catenin (Fig. 5a, b, Supplementary Fig. 5d, e). CP-SkP5 and CP-SkP8 attenuated the interaction between STYK1-GSK3β, whereas CP-SkP2 and CP-SkP6 attenuated the interaction between STYK1-β-catenin (Fig. 5c, d).

We then tested the effects of the disruption peptides on GSK3β sequestration and subsequent Wnt/β-catenin signaling activity. The results showed all of the cell-penetrated peptides decreased the amount of GSK3β within Rab5^+^ endosomes, especially under the condition of Wnt3a treatment (Fig. 5e, f). CP-SkP5, CP-SkP8, CP-SkP2, and CP-SkP6 significantly increased the interaction between GSK3β, Axin1, and β-catenin, and subsequent ubiquitination of β-catenin (Fig. 5g, h). Moreover, the cell-penetrated peptides inhibited the signal of phosphorylated LRP6 S1490 and GSK3β Y216 in the cell membrane fraction upon Wnt3a treatment, which indicated decreased membrane-associated LRP6 signalosomes formation (Fig. 5i). Furthermore, the peptides were found to enhance β-catenin degradation in the CHX chase experiment in PANC-1, AsPC-1, and BxPC-3 cells (Fig. 5j, k, Supplementary Fig. 5f, g). Consistently, the data also showed that CP-SkP5, CP-SkP8, CP-SkP2 and CP-SkP6 inhibited β-catenin/TCF4 transcriptional activity in 7TGC reporter assays and downregulated CyclinD1/Axin2/C-myc expression at both translational and transcriptional levels across multiple PDAC models (PANC-1/BxPC-3/AsPC-1) (Fig. 5l, m, Supplementary Fig. 5h, l). Additionally, all the cell-penetrated peptides reduced the binding affinity of β-catenin with TCF4 (Fig. 5n). Collectively, these results reveal that STYK1-β-catenin or STYK1-GSK3β interaction is essential for STYK1-mediated GSK3β sequestration and subsequent Wnt/β-catenin signaling.

### AP2-mediated STYK1 internalization enhances GSK3β sequestration through autophagy activation

**Fig. 6:**
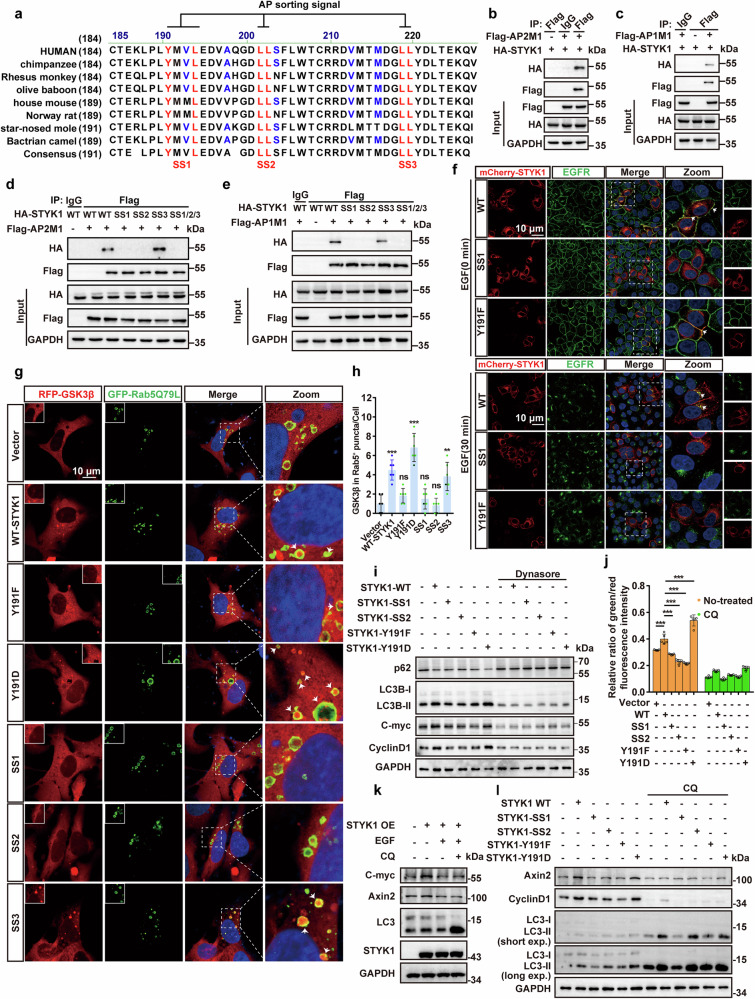
AP2-mediated STYK1 internalization enhances GSK3β sequestration through autophagy activation. **a** Comparative analysis of mammalian STYK1 N-terminal sequences reveals high conservation of two putative AP sorting motifs (highlighted). **b**, **c** The interaction between Flag-tagged AP1/2 μ subunits and HA-tagged STYK1 was analyzed by co-IP. **d**, **e** The interaction between Flag-tagged AP1/2 μ subunits and HA-tagged wild-type STYK1 and its SS1, SS2, and SS3 mutants were analyzed by co-IP using indicated antibodies. **f** Co-localization of mCherry-tagged wild-type STYK1 and its SS1, Y191F mutants with EGFR. Scale bar: 10 μm. **g**, **h** Representative immunofluorescence images of RFP-tagged GSK3β and EGFP-tagged Rab5 Q79L mutant, and the quantification of the amount of GSK3β within Rab5^+^ endosomes upon wild-type STYK1 and its SS1, SS2, SS3, Y191F and Y191D mutants transfection in U2OS cells. Scale bar: 10 μm. **i** Protein levels of autophagy-related genes LC3 and p62, and Wnt target genes(CyclinD1/C-myc/Axin2) in PANC-1 cells with or without Dynasore treatment. **j** The green/red fluorescence intensity ratio of 7TGC signals was quantified in PANC-1 cells transfected with wild-type STYK1 and its mutants (SS1, SS2, SS3, Y191F, and Y191D, *n* = 5). Scale bar: 100 μm. **k** The levels of C-myc, Axin2, and LC3 were analyzed with or without EGF (100 ng/mL), or CQ (100 μM) treatment. **l** The levels of C-yclinD1, Axin2, and LC3 in wild-type STYK1 and its SS1, SS2, SS3, Y191F, and Y191D mutants transfected PANC-1 cell lysates were analyzed with or without CQ (100 μM) treatment. Data were represented as mean ± SD, *p < 0.05; **p < 0.01; ***p < 0.001

Previous studies have shown that clathrin adaptor AP2 facilitates LRP6-signalosome assembly during Wnt/β-catenin pathway activation, highlighting endocytic regulation in mammalian systems.^34,35^ Considering our data that STYK1 plays roles in the membrane-associated LRP6 signalosome and GSK3β sequestration, we therefore asked whether STYK1 is involved in the endocytosis process that regulates Wnt/β-catenin signaling. Bioinformatic profiling of STYK1 revealed three evolutionarily conserved sorting motifs in its N-terminal domain, a tyrosine-based Y191QRL194 and two dileucine-based signals (GDLL204/DGLL220), with the tyrosine motif demonstrating AP1/2 μ-subunit binding specificity (Fig. 6a). As expected, the μ subunit of both AP1 and AP2 were detected in STYK1 immunoprecipitate (Fig. 6b, c). We therefore generated STYK1 proteins with Y191/L194 mutated to alanine (SS1), L203/L204 mutated to alanine (SS2), L199/L220 mutated to alanine (SS3), or all the three mutations (SS1/2/3), and examined the interaction of STYK1 with AP1/2 μ-subunit. The results revealed that either SS1 or SS2, but not SS3 completely blocked the interaction between STYK1 and AP1/2 complex (Fig. 6d, e). We previously reported that STYK1 shifted into cell cytoplasm from cell membrane accompanied with EGFR upon EGF treatment,^15^ Fluorescence microscopy revealed sorting signal mutagenesis disrupted STYK1’s juxtamembrane positioning and EGFR colocalization. Interestingly, SS1 and SS2 mutants prevented STYK1 trafficking to the cell membrane and resulted in endoplasmic reticulum (ER) retention, whereas SS3 showed no effects, suggesting an essential role of the adaptor protein sorting signals of STYK1 in ER-Golgi trafficking (Fig. 6f, Supplementary Fig. 6a, b). In contrast, a single mutant in 191 tyrosine to phenylalanine (Y191F) showed the normal phenotype of ER-Golgi trafficking but significantly inhibited the clathrin/AP2-mediated internalization, indicated by lowered co-localization with EGFR upon EGF treatment compared with wild-type STYK1 (Fig. 6f, Supplementary Fig. 6a). Consistently, the Y191F mutant lowered, whereas the Y191D mutant increased the STYK1-AP2 interaction, but not the STYK1-AP1 interaction (Supplementary Fig. 6c, d). We next investigate the role of STYK1 internalization in regulating the activity of Wnt/β-catenin signaling. The immunofluorescent staining and western blot results showed that STYK1 SS1 and SS2 mutants inhibited GSK3β sequestration (Fig. 6g, h) and significantly decreased the expression of Wnt/β-catenin target genes (Fig. 6i). STYK1 Y191F mutant showed decreased, whereas Y191D showed opposite effects on GSK3β sequestration and Wnt signaling (Fig. 6g–i). Disrupting clathrin/AP2-mediated internalization by dynasore (a cell-permeable dynamin inhibitor) treatment significantly blocked STYK1-mediated activation of Wnt/β-catenin signaling (Fig. 6i).

Autophagy activity was reported necessary for the sequestration of GSK3β,^36^ and our group had proved that STYK1 is a positive regulator of autophagy and plays an essential role in reversing EGF-induced autophagy inhibition by regulating Beclin1 interactome.^15,18^ Disrupting STYK1 membrane trafficking reduced the recruitment of VPS34 to Beclin1, and increased Bcl2 binding with Beclin1 (Supplementary Fig. 6e). We therefore asked whether autophagy activity involved STYK1-induced Wnt/β-catenin signaling. Indeed, the level of p62 and LC3, the most commonly used autophagic flux markers was increased or decreased, respectively upon dynasore treatment (Fig. 6i). EGF-induced autophagy inhibition^19^ and chloroquine (CQ, a lysosomal inhibitor) treatment decreased Wnt signaling in the 7TGC system and down-regulated STYK1-mediated alteration of the Wnt/β-catenin target genes C-myc, Axin2 (Fig. 6j–l, Supplementary Fig. 6f). Furthermore, we further determine the effects of STYK1 on GSK3β sequestration in ATG7^–/–^ human bone osteosarcoma epithelial cells (U2OS cell line). The results indicated that STYK1 failed to enhance the amount of GSK3β within Rab5 Q79L^+^ endosomes upon ATG7 knockout. Additionally, ATG7 knockout also abolished STYK1-mediated expression of the Wnt/β-catenin target genes (Supplementary Fig. 6g, h).

### Phosphorylation of STYK1 at Y191 by BLK kinase enhances pancreatic cancer cell proliferation

**Fig. 7:**
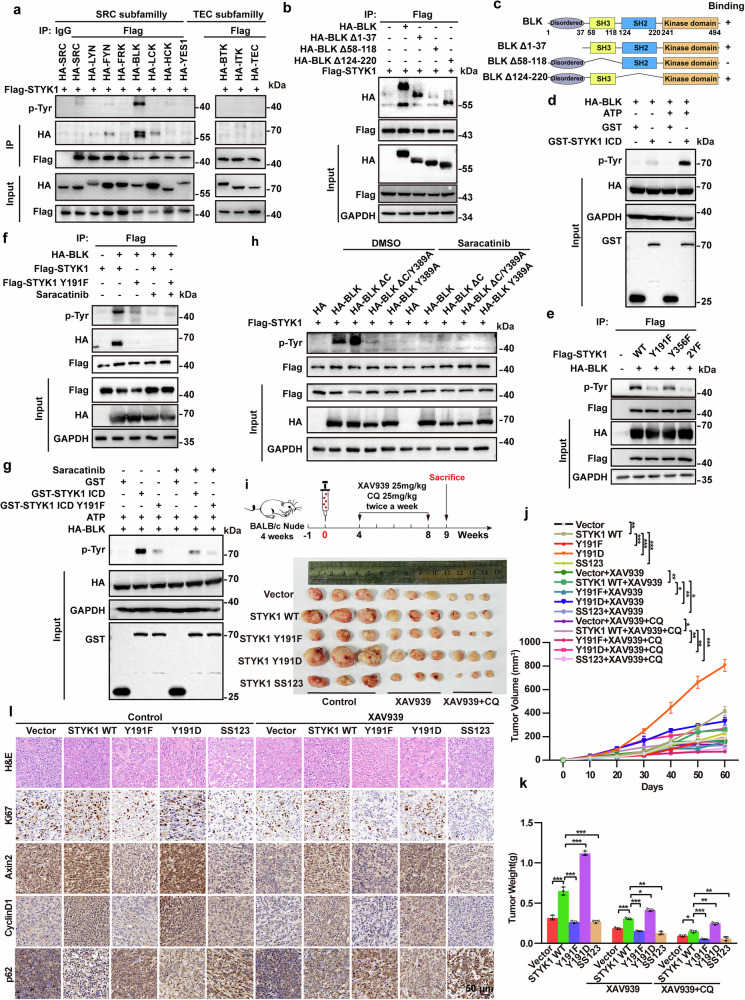
Phosphorylation of STYK1 at Y191 by BLK kinase enhances pancreatic cancer cell proliferation. **a** Co-IP/Western blot analysis of Flag-STYK1 interactions with TEC/SRC kinase subfamily members in HEK293T cells. **b**, **c** Co-IP/Western blot analysis of Flag-STYK1 interactions with HA-tagged BLK mutants in HEK293T cells, with corresponding domain mapping diagrams. **d** GST-STYK1 ICD and HA-BLK kinase reactions (with or without ATP) were analyzed by Western blot using HEK293T lysates. **e** Phosphorylation status of WT STYK1 and Y191F/Y356F mutants in BLK-expressing HEK293T cells was assessed via Western blot. **f** Tyrosine phosphorylation levels of Flag-STYK1 and Y191F mutant in BLK-overexpressing HEK293T cells with or without saracatinib treatment were assessed by IP-Western. **g** GST-STYK1 ICD (WT/Y191F) and HA-BLK kinase reactions (with or without saracatinib/ATP) were analyzed by Western blot using HEK293T lysates. **h** Total phosphorylation levels of wild-type STYK1 upon BLK, its continuous-activated and kinase-dead mutants transfection in HEK293T cells were analyzed using western blot assay. **i** Schematic and representative images of PANC-1 xenograft tumors (Flag-STYK1 WT/Y191F/Y191D/SS123 mutants) with XAV939/CQ treatment timepoints. **j** Tumor growth kinetics post-injection (*n* = 3). **k** Excised tumor weights. **l** H&E and IHC staining (Ki67/Axin2/CyclinD1/p62) in tumor tissues (*n* = 3). Data were represented as mean ± SD, *p < 0.05; **p < 0.01; ***p < 0.001

We have previously reported that STYK1 is phosphorylated at Y191,^18^ and given our data here that the status of Y191 residue is essential for clathrin/AP2 mediated internalization, we next investigated the kinase that is responsible for STYK1 Y191 phosphorylation.^15^ Given EGFR’s limited regulatory effect on STYK1 Y191 phosphorylation despite their direct interaction, subsequent kinase subfamily screening identified BLK (SRC family) as the predominant driver of STYK1 tyrosine phosphorylation and binding potentiation compared to other SRC/TEC members (Fig. 7a). The binding of BLK with purified recombinant GST-tagged STYK1 ICD was also confirmed (Supplementary Fig. 7a). Moreover, we also revealed that the SH3 domain of BLK mediated STYK1-BLK interaction (Fig. 7b, c). Contrary to SH3 domain structural models showing PXXP motif-mediated binding via polyproline II (κ-) helices, functional deletion mapping demonstrated STYK1 interacts with BLK through its kinase domain rather than PXXP motifs (Supplementary Fig. 7b, c). In vitro kinase assays with recombinant GST-STYK1 and HA-BLK immunoprecipitates revealed that STYK1 was indeed phosphorylated at tyrosine residues (Fig. 7d). The tyrosine phosphorylation residue, with evolutionarily conserved Y191 (RTK-subfamily-wide) being critical for BLK-mediated modification, evidenced by Y191F ablation and saracatinib inhibition, unlike Y356F (Fig. 7e–g, Supplementary Fig. 7d, e). Comparative analyses of BLK structural determinants revealed distinct regulatory effects on STYK1 activation. The constitutively active BLK ΔC truncation (lacking the C-terminal inhibitory domain containing autophosphorylation site Y501) demonstrated increased STYK1 phosphorylation, while the catalytically inactive Y389A mutant (ATP-binding deficient) exhibited reduced tyrosine-phosphorylated STYK1 even in the absence of pharmacological inhibition. (Fig. 7h). Additionally, BLK-mediated STYK1 Y191 phosphorylation is essential for β-catenin stability, the binding ability of Axin1 and GSK3β to β-catenin, inhibition of β-catenin ubiquitin and subsequent Wnt/β-catenin target genes expression (Supplementary Fig. 7f–l). These data identify Y191 phosphorylation of STYK1 by BLK as a requisite post-translational modification governing the functional competence of canonical Wnt/β-catenin signaling.

To explore the relevance of STYK1 phosphorylation and AP1/2-mediated STYK1 membrane trafficking in pancreatic cancer cell proliferation, we established isogenic PANC-1 cell lines ectopically expressing wild-type STYK1 or its phosphoregulatory variants (phosphomimetic Y191D, non-phosphorylatable Y191F, and SS1/2/3) by employing a G418-based selection strategy in a STYK1 depletion context. From the in vivo proliferation analysis, we found STYK1 Y191D enhanced tumor growth, while Y191F or SS1/2/3 attenuated progression in PANC-1 xenografts (Fig. 7i–k). Moreover, the Wnt signaling inhibitor XAV939 and autophagy inhibitor CQ both suppressed STYK1-mediated xenograft tumor growth, including mutant variants (Fig. 7i–k). STYK1 Y191D increased, whereas Y191F, SS1/2/3 mutants decreased proliferative activity (Ki67 positive cells) and Wnt targets (Axin2, CyclinD1), but inverse results of the autophagy marker p62 in the IHC analysis (Fig. 7l). These data indicate that Y191 phosphorylation is necessary for STYK1-mediated pancreatic cancer cell proliferation.

### STYK1-driving peptides efficiently inhibit pancreatic cancer development

**Fig. 8:**
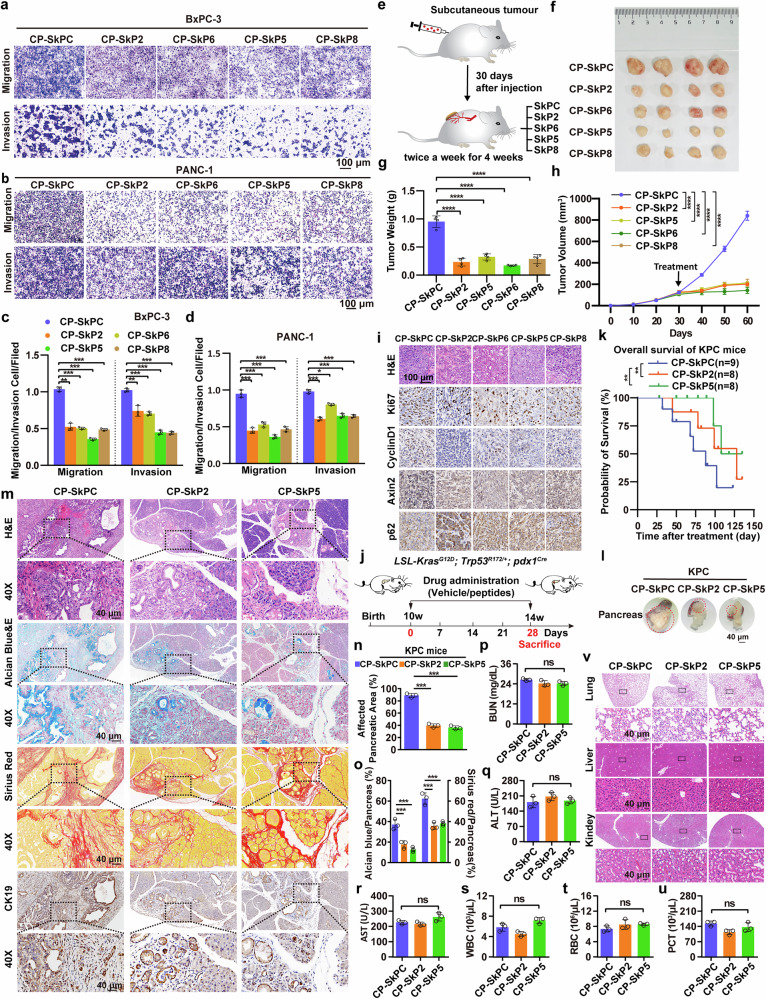
STYK1-driving peptides efficiently inhibit pancreatic cancer development. **a**–**d** Transwell migration/invasion assays in BxPC-3 and PANC-1 cells treated with control or CP-SkP peptides (*n* = 3), including representative images and quantification. Scale bar: 100 μm. **e** Schematic of intraperitoneal peptide administration in nude mouse xenograft models. **f** Excised tumor images from peptide-treated groups. **g** Excised tumor weights. **h** Tumor growth kinetics in peptide-treated mice (*n* = 4). **i** H&E and IHC staining (Ki67/Axin2/CyclinD1/p62) in tumor tissues. Scale bar: 100 μm. **j** Schematic of intraperitoneal peptide administration in KPC mice. **k** Overall survival rates of KPC mice treated with control (*n* = 9), CP-SkP2 (*n* = 8), and CP-SkP5 (*n* = 8) peptides. **l** Representative images of the pancreas in the groups treated with control, CP-SkP2, and CP-SkP5 peptides. Scale bar: 40 μm. **m**, **n** Representative histological images and the quantification of H&E staining in the indicating groups (*n* = 3). Scale bar: 40 μm. **o** The quantification of sirius red or alcian blue staining in the indicating groups (*n* = 3). **p**–**u** The serum concentration of BUN, ALT, AST, WBC, RBC, and PCT in mice treated with control, CP-SkP2, and CP-SkP5 peptides (*n* = 3). **v** Representative H&E images of normal organs including lungs, livers, and kidneys were collected from mice treated with control, CP-SkP2, and CP-SkP5 peptides. Scale bar: 40 μm. Data were represented as mean ± SD, *p < 0.05; **p < 0.01; ***p < 0.001

Given the roles of STYK1 in the activation of Wnt/β-catenin pathway and pancreatic cancer progression, we evaluated STYK1-driving peptides disrupting STYK1-β-catenin/GSK3β interactions on pancreatic cancer tumorigenesis. The results showed that STYK1-driving peptides CP-SkP5, CP-SkP8, CP-SkP2, and CP-SkP6 reduced DNA synthesis and colony formation in PANC-1/BxPC-3 cells (Supplementary Fig. 8a–f), suppressed migration/invasion via wound/transwell assays (Fig. 8a–d, Supplementary Fig. 8g–j). PANC-1 xenograft tumors were further established in nude mice via subcutaneous hindlimb injection (Fig. 8e). 25 mg/kg STYK1-driving peptides were intraperitoneally injected twice a week after the subcutaneous tumor formed. The results revealed that CP-SkP5, CP-SkP8, CP-SkP2, and CP-SkP6 inhibited xenograft growth, downregulating Ki67 and Wnt targets without hepatorenal toxicity (Fig. 8f–I, Supplementary Fig. 8k–q). To further explore the roles of STYK1-driving peptides in pancreatic cancer development, the effects of the peptides in KPC mice were investigated (Fig. 8j). Considering the better effects in the in vitro assays, CP-SkP2 targeting STYK1-β-catenin interaction and CP-SkP5 targeting STYK1-GSK3β interaction were chosen for the following assays. The data showed that CP-SkP2 and CP-SkP5 significantly increased the OS of the KPC mice (Fig. 8k), and reduced tumor size in the pancreata (Fig. 8l). Moreover, histological analysis revealed that CP-SkP2/5 treatment significantly reduced pancreatic lesion burden (PanIN/PDAC replacement) and fibrotic/mucinous markers (sirius red/alcian blue) versus CP-SkPC controls, corroborated by IHC showing diminished CK19^+^ ductal lesions (Fig. 8m, n). Additionally, there were no abnormalities in the kidney or liver function and no morphological changes in normal functional organs as well (Fig. 8o–v). Collectively, these data indicate that STYK1-driving peptides show efficient effects in inhibiting pancreatic cancer development.

## Discussion

While the Wnt/β-catenin pathway plays a critical role in driving PDAC progression,^24^ however, genetic alterations in core pathway components like β-catenin and APC occur infrequently. This disconnect highlights significant gaps in understanding how this signaling axis sustains persistent activation in PDAC pathogenesis, a mechanistic puzzle that remains poorly characterized. In the present study, we reported that higher STYK1 expression correlates with poor pancreatic cancer survival and STYK1 deletion alleviates pancreatic cancer progression using *LSL-Kras*^*G12D/+*^; *Trp53*^*R172H/+*^; *Pdx1*^*Cre*^; *Styk1*^*−/−*^ mice (KPCS mice). Mechanically, we reported that STYK1 directly interacts with β-catenin and GSK3β, disrupting destruction complex formation and thereby preventing β-catenin ubiquitination to enhance its stability. We also demonstrated that STYK1 promotes cell membrane-associated GSK3β sequestration into MVBs, which results in the physical separation of GSK3β from its cytoplasmic targets and negative regulation of GSK3β in an ESCRT-dependent manner. Moreover, we showed that the phosphorylation of STYK1 at Y191 by BLK kinase promotes clathrin/AP2-mediated internalization of STYK1 and membrane-associated GSK3β sequestration. Disrupting STYK1-β-catenin or STYK1-GSK3β interaction significantly inhibits GSK3β sequestration, subsequent Wnt/β-catenin signaling, and pancreatic cancer development (Supplementary Fig. 9).

The activation of the canonical Wnt/β-catenin pathway upon Wnt stimulation mainly depends on the inactivation of GSK3β. Regulation of GSK3β is tightly controlled through a series of direct and indirect aspects. Specifically, autophosphorylation at Y216 locks the kinase in its active state, while phosphorylation at S9 acts as a molecular brake by disabling its catalytic capacity. Beyond these biochemical switches, cells employ indirect strategies, either trapping GSK3β in cytoplasmic protein assemblies that obstruct substrate interactions or sequestrating it into multivesicular bodies via Wnt signaling, effectively cutting off its access to cytosolic targets like β-catenin.^11^ However, the prevailing view positioning GSK3β compartmentalization as a mandatory checkpoint for Wnt pathway activation remains contentious within the field, with ongoing debates highlighting persistent gaps in understanding the mechanistic underpinnings. For example, constitutively active N-terminally truncated LRP6-induced chronic Wnt hyperactivation may trigger compensatory damage-limitation mechanisms not engaged during physiological Wnt signaling in development or tissue homeostasis.^37^ Our data here showed that Wnt activation using Wnt3a treatment also supports the separation of GSK3β into MVBs. Moreover, Zeng et al showed that GSK3β is required for LRP6 phosphorylation and proposed cytosolic GSK3β-CK1α complexes phosphorylate β-catenin to suppress Wnt/β-catenin signaling, while membrane-localized GSK3β-CK1α phosphorylates LRP6 to activate the pathway during Wnt stimulation.^32^ The plasma and membrane separation experiment in the current study showed that membrane-associated GSK3β and its kinase-active form GSK3β Y216 were significantly increased along with phosphorylated LRP6 Y1490. Moreover, the level of phosphorylated GSK3β Y216 was also increased in the LRP6 immunoprecipitate upon Wnt activation.

Receptor-mediated endocytosis represents a highly selective cellular mechanism through which macromolecular cargo (e.g., ligand-bound receptor assemblies) undergoes regulated internalization. Clathrin- (CME) and caveolae-mediated (CavME) endocytosis, the two predominant pathways, has been extensively studied for regulating receptor trafficking across signaling (EGFR), transport (transferrin), and adhesion (integrins/cadherins) systems.^38^ In CME, dynamically assembled scaffolding complexes coordinate membrane curvature generation and scission of the coated vesicle through sequential protein recruitment. CavME operates through caveolin-1-enriched curved membrane domains that selectively concentrate cholesterol-rich membrane microdomains.^38^ Caveolin-mediated endocytosis has been suggested to promote β-catenin stabilization and potentiate Wnt signaling, while LRP6 intracellular tyrosine phosphorylation disrupts caveolin-rich lipid raft signalosome assembly, thereby attenuating pathway activation.^39,40^ On the other hand, emerging evidence implicates clathrin-dependent trafficking in modulating Wnt pathway dynamics.^12,41^ For example, studies reveal that PtdIns(4,5)P(2)-dependent recruitment of clathrin-AP2 complexes drives LRP6 signaling hub assembly at membranes, where disrupting these components prevents signalosome organization.^42^ Further investigations demonstrate that the Ap2μ2 subunit interacts with DVL2 and stabilizes this effector during internalization. Impairing Ap2μ2 triggers DVL2 proteolysis, blocks membrane-proximal signalosome assembly, and ultimately dampens β-catenin-dependent transcriptional output.^34^ SNX27, a regulator of clathrin-mediated endocytosis, interacts with Fzd7 to promote its internalization and degradation, thereby suppressing TCF/LEF transcriptional activity.^43^ Both caveolin- and clathrin-based mechanisms in Wnt pathway activation were assumed to be dependent on cell type with APC acting as the “gatekeeper”.^44^ Our data showed that clathrin/AP2-mediated STYK1 is also important for GSK3β sequestration and subsequent Wnt/β-catenin activation.

Macroautophagy (hereafter referred to as autophagy), a cellular self-digestion program where double-membrane autophagic vesicles traffic cytoplasmic cargo to lysosomal compartments for recycling, serves as a key driver in pancreatic oncogenesis.^45,46^ However, autophagy was reported to negatively regulate Wnt signaling by promoting Dishevelled and β-catenin protein degradation.^47,48^ Thus, the synergistic mechanism of autophagy and Wnt/β-catenin in promoting the development of pancreatic cancer remains extremely unclear. To answer this question, our group has revealed that lncRNA PVT1 acts as the notion that links both activation of autophagy and Wnt/β-catenin signaling through modulating the expression of Pygo2 and ATG14, the specific regulator of TCF/LEF transcription factor and autophagy PtdIns3K-C1 complex respectively.^49^ We also showed that the β-catenin/TCF4 complex transcriptionally upregulates TSPAN1, a key autophagy maturation regulator.^20^ As we revealed that STYK1 facilitated autophagy activity through directly binding to the PtdIns3K-C1 complex and elevating the serine phosphorylation of BECN1 to disrupt BECN1-BCL2 interaction,^18^ here we demonstrated a new mechanism that STYK1 accelerated Wnt/β-catenin signaling through AP2 mediated separation of GSK3β into MVBs and direct inhibition of GSK3β for the cooperation of autophagy and Wnt/β-catenin pathway in pancreatic cancer progression.

BLK primarily functions in B lymphocytes and developing thymocytes, experimental activation of this kinase in mouse T-cell precursors also drives malignant transformation, ultimately triggering aggressive T-cell lymphoma development.^50^ Preclinical models consistently reveal that sustained BLK activation accelerates lymphomagenesis by driving neoplastic progression.^51^ Emerging data now extends BLK’s oncogenic portfolio to epithelial tumors, with pancreatic adenocarcinoma showing particular vulnerability.^52^ Elevated BLK kinase activity has been shown to activate PDX1-mediated transcriptional networks, promoting excessive insulin production and suggesting its potential as a therapeutic target in pancreatic cancer.^53^ Our previous study also defined that FAM83A could be phosphorylated by BLK kinase the phosphorylation revealed an oncogenic function in pancreatic cancer progression.^25^ Consistently, here we screened and recognized BLK as the upstream kinase for STYK1 phosphorylation at Y191 residue, which is essential for the binding with AP2 and clathrin/AP2-mediated internalization, and enhanced the development of pancreatic cancer. Inflammation is paramount in pancreatic oncogenesis. γδT cells are a non-major histocompatibility complex (MHC)-restricted lymphocyte subset. γδT cells were reported to produce high levels of tumor-promoting interleukin-17 (IL-17) in pancreatic cancer (Tγδ17 cells).^54^ Deletion, or blockade of Tγδ17 cell recruitment was protective against pancreatic cancer progression.^55^ BLK-deficient mice revealed an essential role in the development of Tγδ17 cells.^56,57^ Moreover, STYK1 was reported to be specifically expressed γδT cells.^58^ Whether STYK1 or BLK-induced phosphorylation status functions in the tumor-promoting γδT cells and its relevance with STYK1-mediated intracellular signal cascade in pancreatic cancer development need further exploration in the future.

Continuously activated Wnt/β-catenin signaling via genetic or epigenetic ways, serves as a potent oncogenic driver across malignancies, spurring intensive efforts to develop targeted inhibitors. Nonetheless, tissue homeostasis and regeneration could be impaired upon the blockade of Wnt signaling, which needs to be resolved and limits Wnt-based therapies. Recent breakthroughs have unmasked tumor-specific vulnerabilities, identifying cancer-cell-restricted regulators that fine-tune Wnt signaling outputs, offering promising avenues for selectively crippling oncogenic Wnt activity.^59^ For example, secreted frizzled-related proteins antagonize Wnt signaling by ligand sequestration, demonstrating tumor-suppressive effects in preclinical models through their peptide derivatives.^60^ v-ATPase upregulation drives Wnt/β-catenin-dependent tumorigenesis. Targeting v-ATPase using its inhibitors, such as bafilomycin and concanamycin, showed marked inhibition of Wnt/β-catenin signaling in colorectal cancer and proposed the blockade of v-ATPase as a viable option for colorectal cancer treatment.^61^ We previously also revealed that FAM83A-targeting peptides destabilize β-catenin and suppress the malignant properties of pancreatic cancer cells.^25^ Consistently, here we further defined the inhibitory role of STYK1-derived peptides in pancreatic cancer development. However, the translational potential of STYK1-targeting peptides necessitates cautious evaluation of potential limitations, including off-target effects due to sequence homology with other tyrosine kinase motifs and inter-patient variability in STYK1 dependency. Ongoing studies utilizing patient-derived xenograft models and phosphoproteomic profiling aim to delineate biomarker-driven stratification strategies while assessing therapeutic specificity. Collectively, our findings establish STYK1 as a modulator of Wnt/β-catenin signaling and confirm its therapeutic targeting potential in pancreatic cancer.

## Materials and methods

### Animal studies

All animal experiments were performed following the guidelines and regulations and approved by the Hubei University of Technology Animal Care and Use Committee. (1) Generation of KPCS mice: *Styk1* knockout mice were generated by Cyagen Biosciences, Inc. (Shanghai, China). Briefly, CRISPR/Cas9-mediated *Styk1* exon2-4 knockout in embryonic stem cells (ESCs) generated blastocyst-derived chimeras that were crossed to *LSL-Kras*^*G12D/+*^; *LSL-Trp*^*53R172H/+*^; *Pdx1*^*Cre*^ mice to generate *LSL-Kras*^*G12D/+*^; *LSL-Trp*^*53R172H/+*^; *Pdx1*^*Cre*^; *Styk1*^*−/−*^ (KPCS) pancreatic cancer models with genotyping confirmed by PCR. PCR primers used for analyzing were listed: Styk1: F: 5’-CAGTGGATGCGGCTCAGTTGGTAG-3’; R: 5’-ATTTGTTTTCCAGGCCAGGC-3’. Pdx-Cre: F: 5’-CCTGGACTACATCTTGAGTTGC-3’; R: 5’-AGGCAAATTTTGGTGTACGG-3’. Kras: F1: 5’-GCAGGTCGAGGGACCTAATA-3’; F2: 5’- CTGCATAGTACGCTATACCCTGT-3’; R: 5’-TGTCTTTCCCCAGCACAGT-3’. Trp53: F1: 5’-CCATGGCTTGAGTAAGTCTGCA-3’; F2: 5’-GAAACTTTTCACAAGAACCAGATCA-3’; R: 5’-AGGTGTGGCTTCTGGCTTC-3’. (2) Mouse subcutaneous xenograft experiments were carried out as previously described.^25^ Female BALB/c nude mice (4-week-old, 18–22 g) were sourced from Hunan SJA Laboratory Animal (Cat: hnslkjd006; Changsha, China) and housed under SPF conditions with routine health surveillance. Briefly, Female BALB/c nude mice were subcutaneously implanted with PANC-1 xenografts (3 × 10^6^ cells/axilla) and treated biweekly via intraperitoneal injection with XAV939 (Wnt inhibitor), CQ (autophagy inhibitor), or synthetic peptides (25 mg/kg), showing reduced tumor growth quantified by caliper-measured volume (*V* = length × width^2^/2) and final excised tumor weights.

### Specimen and ethical statement

Human PDAC tissues used in this study were obtained with approval from the Ethics Committee of Zhongnan Hospital of Wuhan University (Approval No.: 2023165 K). Written informed consent was obtained from all participants enrolled in the study. Tissue samples were collected from patients with pancreatitis or PDAC who underwent surgery or biopsy at Zhongnan Hospital of Wuhan University. The collected samples were subsequently paraffin-embedded, sectioned, and subjected to immunofluorescence staining.

### Western blot

Whole-cell extracts were prepared with 1% sodium dodecyl sulfate lysis buffer with freshly added 1% proteinase inhibitor cocktail (Biomake, B14001) and 1 mM phenylmethylsulfonyl fluoride (PMSF, MCE, HY-B0496). Western blot was performed as described previously.^20^ The following primary antibodies were used: anti-phospho-β-catenin (Ser33/37/Thr41) (9561, Cell Signaling Technology), anti-β-catenin (51067-2-AP, Proteintech), anti-β-TrCP (4394, Cell Signaling Technology), anti-Non-phospho (Active) β-catenin (Ser33/37/Thr41) (8814, Cell Signaling Technology), anti-STYK1 (862318, zenbio), anti-C-myc (10828-1-AP, Proteintech), anti-CyclinD1 (60186-1-Ig, Proteintech), anti-Axin2 (20540-1-AP, Proteintech), anti-mouse HA (M180-3, EMD Millipore), anti-Rabbit HA (51064-2-AP, Proteintech), anti-mouse DYKDDDDK (M185, MBL), anti-Rabbit DYKDDDDK (80010-1-RR, Proteintech), anti-GAPDH (60004-1-Ig, Proteintech), anti-GFP (598, EMD Millipore), anti-phosphotyrosine (P4110, Sigma), anti-GSK3β (22104-1-AP, Proteintech), anti-GSK3β S9 (9336, Cell Signaling Technology), anti-GSK3β Y216 (ab68476, Abcam), anti-Axin1 (16541-1-AP, Proteintech), anti-BLK (10510-1-AP, Proteintech), anti-Lamin B1 (12987-1-AP, Proteintech), anti-VPS24 (15472-1-AP, Proteintech), anti-HRS (10390-1-AP, Proteintech), anti-TCF4 (22337-1-AP, Proteintech), anti-LC3B (381544, zenbio), anti-p62 (66184-1-Ig, Proteintech), anti-EEA1 (3288, Cell Signaling Technology), anti-CD63 (MEM-259, Thermo Fisher), anti-LRP6 (sc-25317, Santa Cruz), anti-LRP6 S1490 (2568S, Cell Signaling Technology), anti-LBPA (MABT837, Sigma-Aldrich).

### GST affinity-isolation assay and surface plasmon resonance

GST affinity-isolation assay was performed as described previously.^18^ The cDNA encoding STYK1 ICD (aa 26–422), β-catenin, or GSK3β was cloned into pGEX4T-1 (GE, 27-4580-01). The cDNAs encoding β-catenin or GSK3β were cloned into pET-28a (EMD Biosciences, 69864-3). GST-tagged and His-tagged fusion proteins were incubated at 4 °C using glutathione-sepharose beads or NI-sepharose beads. GST fusion protein was eluted with reduced glutathione. Purified GST-tagged STYK1 protein was incubated with His-tagged β-catenin or GSK3β fusion protein and NI-sepharose beads overnight at 4 °C. Expression of GST fusion proteins was confirmed by western blot assay. Surface plasmon resonance analysis (Biacore T200) quantified binding kinetics between GST-β-catenin/GSK3β and synthetic α-helical peptides, with dissociation constants calculated via BIA evaluation software.

### Statistical analysis

Animal studies utilized *n* = 3–6 mice per group with randomized allocation, while in vitro experiments were performed in triplicate with independent biological replicates. All statistical analyses were performed using GraphPad Prism 6.0 software (GraphPad, La Jolla, CA, USA). Triplicate data are expressed as mean ± SD, with significance (*p* < 0.05, *p* < 0.01, *p* < 0.001) determined by two-tailed t-test (two groups) or ANOVA (multiple groups). *represents *p* < 0.05, **represents *p* < 0.01, and ***represents *p* < 0.001.

## Supplementary information

Supplementary materials and figures

## Data Availability

The data supporting this study are available within the paper and its Supplementary Data file. The RNA sequencing data in this study have been deposited in NCBI’s Gene Expression Omnibus (GEO) under accession code GSE298057.
