# Supplementary material for: Serine/threonine/tyrosine kinase 1 drives pancreatic carcinogenesis via GSK3β sequestration-mediated Wnt/β-catenin pathway hyperactivation
Source: Signal Transduct Target Ther. 2025 Jun 30;10:205. doi: 10.1038/s41392-025-02292-x (PMC12209456; doi:10.1038/s41392-025-02292-x)
Supplement: Supplementary file 1 — Supplementary materials and figures [file 41392_2025_2292_MOESM1_ESM.docx]

Supplementary Materials for

Serine/threonine/tyrosine kinase 1 drives pancreatic carcinogenesis via GSK3β sequestration-mediated Wnt/β-catenin pathway hyperactivation

Cefan Zhou^1,2#^, Xueying Dong^1#^, Shi Li^1#^, Yue Xi^1^, Yuan Liu^1^, Xuehong Qian^1^, Ziyan Song^1^, Li Zhou^3^, Rui Zhang^1^, Hao Lyu^1^, Shuai Xiao^1^, Dong Guo^1^, Qi Zhang^1^, Weiyong Liu^4^, Yan Xiong^5^, Zhentian Wang^6^, Chaojun Yan^1^, Zijian Zhang^1^, Haichuan Zhu^1^, Xing-Zhen Chen^2^, Zhiyin Song^7^ and Jingfeng Tang^1, 2, 8*^

Correspondence to: [Jingfeng_hut@163.com](mailto:Jingfeng_hut@163.com)

**This PDF file includes:**

Materials and Methods

Figures. S1 to S9

**Materials and methods**

## Cell lines and reagents

Human pancreatic cancer cell lines PANC-1 (TCHu 98), AsPC-1 (TCHu 8), SW1990 (TCHu201), MIA-PACA-2 (TCHu271), and BxPC-3 (TCHu 12) were purchased from the cell center of the Institute of Biochemistry and Cell Biology, Chinese Academy of Sciences (Shanghai, China). SW1990 was purchased from Cobioer (Nanjing, China). HEK293T, U2OS, and HeLa cells were stored in our lab. Cells were cultured in Dulbecco’s modified Eagle’s medium (DMEM) (Gibco, USA) medium supplemented with 10% fetal bovine serum (Gibco, USA), 100 U/ml penicillin G, and 100 μg/ml streptomycin at 37°C in a humidified incubator containing 5% CO2. Wnt3a was purchased from R&D systems (5036-WN). Wnt inhibitor XAV939 and BLK kinase inhibitor saracatinib were purchased from MedChemExpress (HY-15147, HY-10234). Autophagy inhibitor chloroquine diphosphate salt (CQ) was purchased from Sangon Biotech (A506569). Cycloheximide (CHX) and MG132 were purchased from MedChemExpress (HY-12320 and HY-13259).

## Plasmids, siRNAs and transfection

DNA fragments encoding BLK, SRC, BTK, ITK, TEC, LYN, FYN, FRK, LCK, HCK, and YES1 amplified by PCR were gifts from Prof. Jiahuai Han (Xiamen University, China) and cloned into pcDNA3.1-3×HA. The PCR products of STYK1, GSK3β, and Axin1 were cloned into pcDNA3.1-3×HA, pCMV-3×Flag (Sigma, E4401), and pCMV-mCherry (constructed by our lab) respectively. The PCR products of β-catenin were cloned into pCMV-N1-GFP (Clontech, 6085-1). To make STYK1, β-catenin, GSK3β, or BLK mutants or truncations, a series of forwarding primers harboring the desired mutations were used in PCRs. The nucleotide sequences of all constructs were confirmed by DNA sequencing. 7TGC was a gift from RoelNusse (Addgene, 24304). DNA and interference RNA were transfected with Lipofectamine 3000 (Invitrogen, L3000015) according to the manufacturer’s instructions. shRNA for STYK1 #1: 5ʹ- CAAGTATATCACATCGGAAAGCTCGAGCTTTCCGATGTGATATACTTG -3ʹ. #2: 5ʹ- GCCCATCTTTCGAGCCAATATCTCGAGATATTGGCTCGAAAGATGGGC -3ʹ. siRNA for STYK1 was 5ʹ- CCAUCUUUCGAGCCAAUAUTT -3ʹ. siRNA for β-catenin was 5ʹ- AGGUGCUAUCUGUCUGCUCUA -3ʹ. siRNA for HRS was 5ʹ- CCGGAGTCTGATGTGAACTAA -3ʹ, siRNA for VPS24 was 5ʹ- CCTCCCAAAGAGCTGGTTAAT -3ʹ.

## Co-immunoprecipitation (Co-IP)

Co-IP analysis was performed as described before.[^1^](#_ENREF_1) Briefly, cells were harvested and lysed in RIPA lysis buffer (50 mM Tris-HCl pH 7.5, 150 mM NaCl, 1% Triton X-100 [Sangon Biotech, 9002-93-1], 10 mM NaF, and 2 mM EDTA) containing proteinase inhibitor cocktail (Biomake, B14001) and Halt phosphatase inhibitor cocktail (Thermo Fisher Scientific, 78420). The supernatants of cell lysates were incubated overnight with primary antibodies according to each experiment after pre-cleared with protein A/G magic beads (Bimake, B23202), and then incubated with protein A/G magic beads for 2 h at 4°C. The immunoprecipitate was then subjected to western blotting.

## Immunohistochemical (IHC)

Pancreatic tumor tissues were sectioned at a thickness of 3.5 μm and performed with Hematoxylin and eosin (H&E) staining according to the manufacturer's instructions. The distinguishing of the normal pancreas, early PanIN (ADM and PanIN1), late PanIN (PanIN2 and PanIN3), and PDAC were classified according to the standard described previously [^2^](#_ENREF_2). Tissue sections were blocked with 5% normal goat serum with 0.1% Triton X-100 and 3% H_2_O_2_ in PBS for 60 min at room temperature and then incubated with anti-CK19 (GB11197, Servicebio), anti-C-myc (10828-1-AP, Proteintech), anti-CyclinD1 (60186-1-Ig, Proteintech), anti-Axin2 (20540-1-AP, Proteintech), anti-LC3 (14600-1-AP, Proteintech), anti-p62 (66184-1-Ig, Proteintech) overnight, which were then detected by HRP-conjugated DAB.

## Immunoﬂuorescence

PANC-1, AsPC-1, HEK293T, or HeLa cells were ﬁxed with 4% formaldehyde for 10-15 min and then blocked with 3% BSA and 0.1% Triton X-100 in PBS for 20 min at room temperature. Immunostaining was performed using the indicated antibody. Nuclei were counterstained with DAPI. Images were taken with a Leica Inverted Confocal SP8 (Leica, Germany).

## Alcian blue and sirius red staining

Pancreas sections were deparafﬁnized, hydrated, and immersed in alcian blue (Vector lab, H-3501) solution for 30 min at room temperature. The slides were counterstained in nuclear fast red or eosin after washing with distilled water. Strongly acidic mucosubstances will be stained blue. collagen was stained with Picro-Sirius red (Sigma, S365548). After staining, slides were washed in two dips of acidified water and dehydrated with 100% ethanol. Finally, slides were cleared in xylene and mounted.

## RNA extraction and qRT-PCR

Total RNA was extracted from cultured pancreatic cancer cells using Trizol reagent (Invitrogen) according to the manufacturer’s protocol. qRT-PCR was performed as previously described.[^3^](#_ENREF_3) The mRNA expression level for each sample was normalized to the expression of GAPDH using the 2 ^-ΔΔct^ method[^4^](#_ENREF_4) with three biological replicates of comparative qRT-PCR. The following primer sequences were used for qRT-PCR: GAPDH, (forward) 5′- AGCCACATCGCTCAGACAC -3′ and (reverse) 5′- GCCCAATACGACCAAATCC -3′; CyclinD1, (forward) 5′- GCTGCGAAGTGGAAACCATC -3′ and (reverse) 5′- CCTCCTTCTGCACACATTTGAA -3′; C-myc, (forward) 5′- CCTGGTGCTCCATGAGGAGAC -3′ and (reverse) 5′- CAGACTCTGACCTTTTGCCAGG -3′; Axin2, (forward) 5′- TACACTCCTTATTGGGCGATCA -3′ and (reverse) 5′- TTGGCTACTCGTAAAGTTTTGGT -3′; STYK1, (forward) 5′- TCGAGCCAATATGAACACTGGG -3′ and (reverse) 5′- TCGCCCTAAGAAATCTTGTACCT -3′. Amplification was performed in an ABI Q3 real-time PCR system using a SYBR Green master mix (YEASEN, 11201ES08).

## Lentiviral production and creation of stable cell lines

STYK1 shRNAs were subcloned into the lentiviral vector pLKO.1-puro (Sigma, 8453), which were co-transfected with viral packaging plasmids psPAX2 and pMD2.G into 293T cells in 10 cm dishes. Then the viral supernatant was harvested at 48 h post-transfection and filtered through a 0.22 µm membrane. After applying the viral supernatant to PANC-1 or AsPC-1 cells with 10 μg/μl of polybrene (Solarbio, H8761), selection for puromycin (Solarbio, IP1280) and/or G418 (Yeasen, 60220ES03) resistance was initiated 48 h after transfection. The selection medium was changed every 3-4 d for several weeks, and clones of puromycin and/or G418-resistant cells were isolated and expanded for further characterization.

## TOP/FOP ﬂash assay

M50 Super 8x TOPFlash and M51 Super 8x FOPFlash (TOPFlash mutant) were gifts from Randall Moon (Addgene, 12456 and 12457). The human pancreatic cancer PANC-1, AsPC-1, or HEK293T cells were transfected with the M50 and M51plasmids along with an internal Renilla control plasmid. The luminescence ratio of the experimental reporter (Firefly) to the control reporter (Renilla) was calculated. All experiments were performed in triplicate.

## 5-Ethynyl-20-deoxyuridine (EdU) incorporation assay

EdU labeled PANC-1 and AsPC-1 cells stably expressing sh-STYK1 or PANC-1 and BxPC-3 cells treated with CP-SkP5, CP-SkP8, CP-SkP2, and CP-SkP6 peptides at 5μM for 24 h were examined with the BeyoClick™ EdU Cell Proliferation Kit with Alexa Fluor 555 or 488 (Beyotime, C0075S, C0071S). Cells were photographed under an Olympus FSX100 microscope.

## Wound-healing assay

PANC-1 and BxPC-3 cells were placed in twelve-well plates and grown until 90% confluence. A straight wound through the cell layer was made with a sterile pipette tip. Then, Cells were treated with CP-SkP5, CP-SkP8, CP-SkP2, and CP-SkP6 peptides at 5μM and incubated for 12 h or 36 h. Cell migration was analyzed via the wound closure ratio.

## Impedance-based real-time cell analysis (RTCA)

The rate of cell proliferation was monitored in real-time using the xCELLigence system (E-plate and E-insert). Totally 1×10^4^ PANC-1 or AsPC-1 cells stably expressing sh-STYK1 were seeded in the E-plate. Data were collected and the impedance value was automatically monitored by the xCELLigence system for indicated times and expressed as a Cell index (CI) value.

## Trans-well assays

PANC-1 and BxPC-3 cells were placed in the upper well of an uncoated trans-well chamber with an 8-µm φ pore membrane. DMEM culture medium containing 20% FBS and treated with CP-SkP5, CP-SkP8, CP-SkP2, and CP-SkP6 peptides at 5μM for 24 h were then added to the lower chamber of the trans-well chamber. The plates were incubated at 37 °C for 24 h. After the removal of all nonmigrated cells, the migrated cells were stained using crystal violet. A microscope was employed to capture the images and count the migrated cells.

## Colony formation assay

Colony formation assay was performed as described previously.[^5^](#_ENREF_5) human pancreatic cancer BxPC-3 cells were seeded into a 24-well plate and incubated with a complete medium at 37 °C for 2 - 3 weeks. The cells were fixed with 4% paraformaldehyde and stained with 2% crystal violet after treatment with CP-SkP5, CP-SkP8, CP-SkP2, and CP-SkP6 peptides at 5μM concentration. The images were obtained and the number of colonies was counted. Each experiment was repeated in triplicate.

## RNA-sequencing

Total RNA was extracted from control and shSTYK1 PANC-1 cells (three biological replicates per group) using Trizol reagent. The mRNA was then purified using Dynabeads Oligo (dT) (Thermo Fisher Scientific) and randomly cleaved into fragments approximately 300 bp long. These fragments were inverted to synthesise cDNA, which was then ligated to an adaptor. The products were purified, fragmented and PCR amplified to obtain the final library, which was sequenced on the NovaSeq X Plus platform. The raw data has been uploaded to NCBI’s Gene Expression Omnibus (GEO) under accession code GSE298057.

**
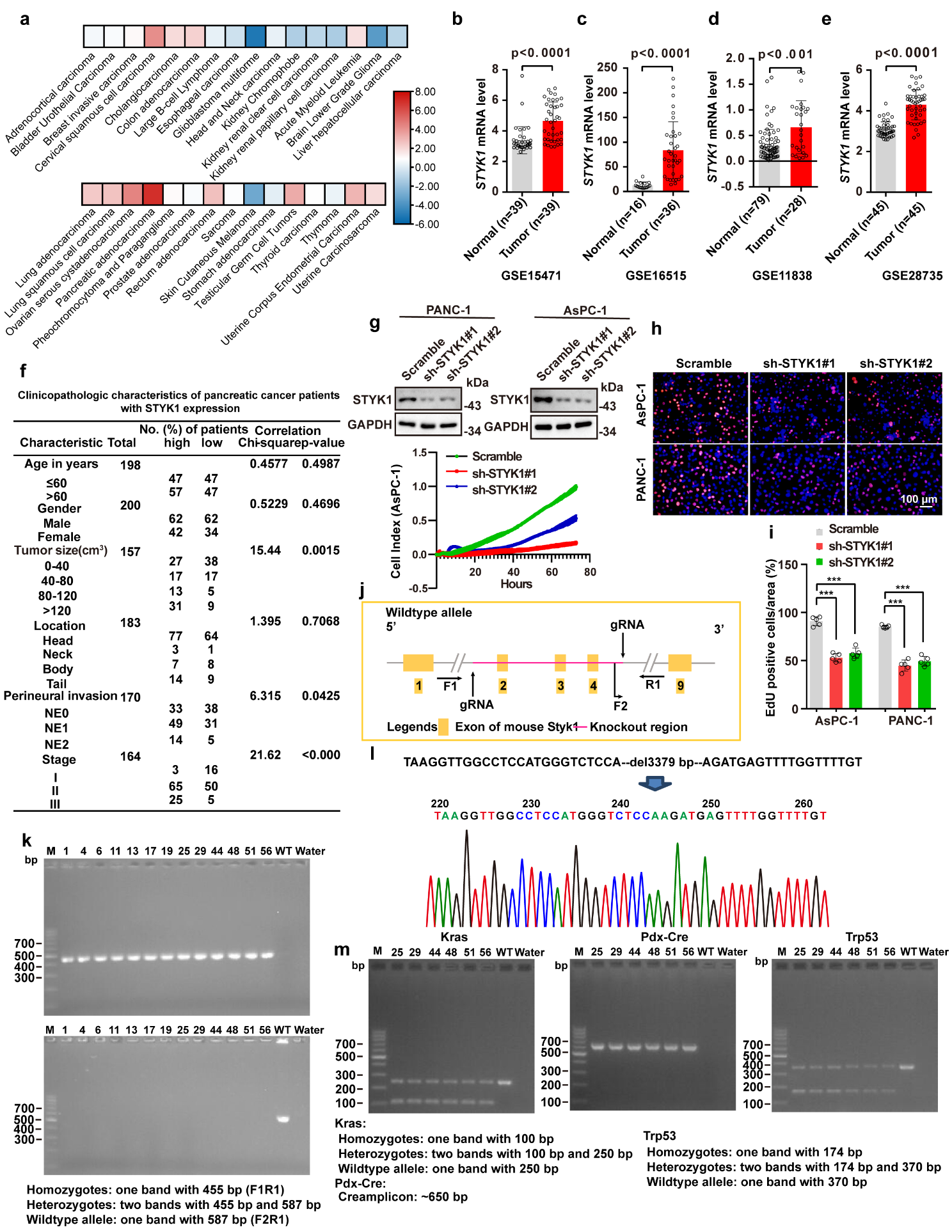
Supplementary Figure 1.** STYK1 is upregulated and STYK1 deletion alleviates pancreatic cancer progression, related to Fig.1.

**a-e** Relative *STYK1* mRNA level in GEPIA2 database and pancreatic cancer gene microarray dataset (GSE15471, GSE16515, GSE11838, GSE28735). **f** Clinicopathologic characteristics of pancreatic cancer patients with STYK1 expression. **g** The efficiency of STYK1 depletion upon stable expression of STYK1 shRNAs in PANC-1 and AsPC-1 cells and impedance-based real-time cell analysis showing the differences in growth proﬁle for AsPC-1 cells upon STYK1 knockdown measured in a 16-well E-plate. **h, i** DNA synthesis ability of the cells with or without STYK1 depletion was assessed by EdU assays in PANC-1 and AsPC-1 cells. Scale bar: 100 μm. **j-m** The diagram of the strategy for the generation of Styk1 KO mice and verification for the genotyping using gel electrophoresis and genomic sequencing assays. Data were represented as mean ± SD, **p* < 0.05; ***p* < 0.01; ****p* < 0.001.

**
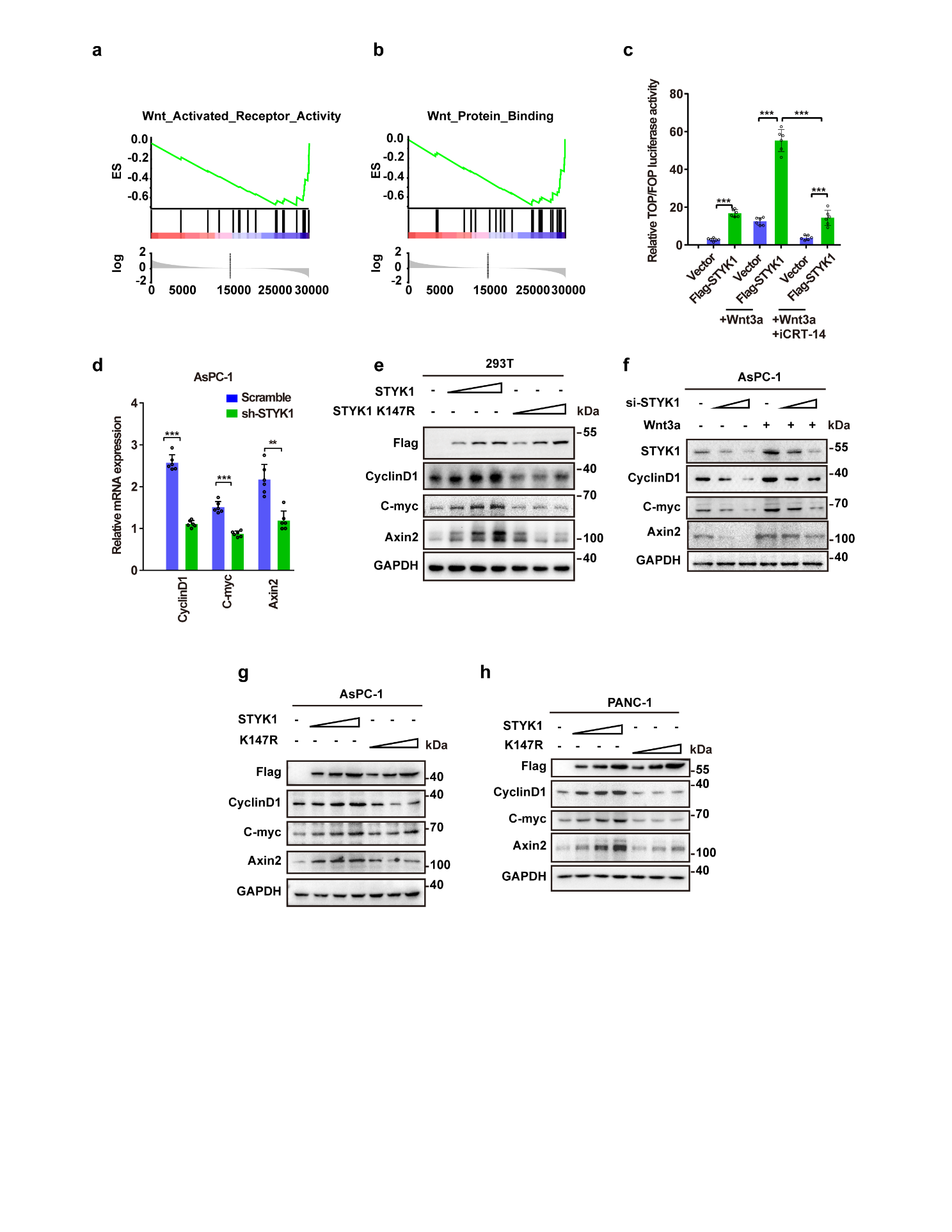
**

**Supplementary Figure 2.** STYK1 promotes canonical Wnt/β-catenin signaling, related to Fig.2.

**a, b** GSEA analysis of enriched gene set in the Wnt activated receptor activity and Wnt related protein binding comparison of TCGA pancreatic cancer database with distinguishing STYK1 level. **c** Relative Wnt reporter luciferase activity (TOP/FOPflash) in HEK293T cells with or without Wnt3a or iCRT-14 treatment. **d-h** mRNA and protein levels of Wnt target genes CyclinD1, C-myc and Axin2 in PANC-1, AsPC-1 and HEK293T cell lysates upon STYK1 depletion, or overexpression of wild-type STYK1 or its K147R mutant with or without Wnt3a treatment. Data were represented as mean ± SD, *P < 0.05; **P < 0.01; ***P < 0.001.

**
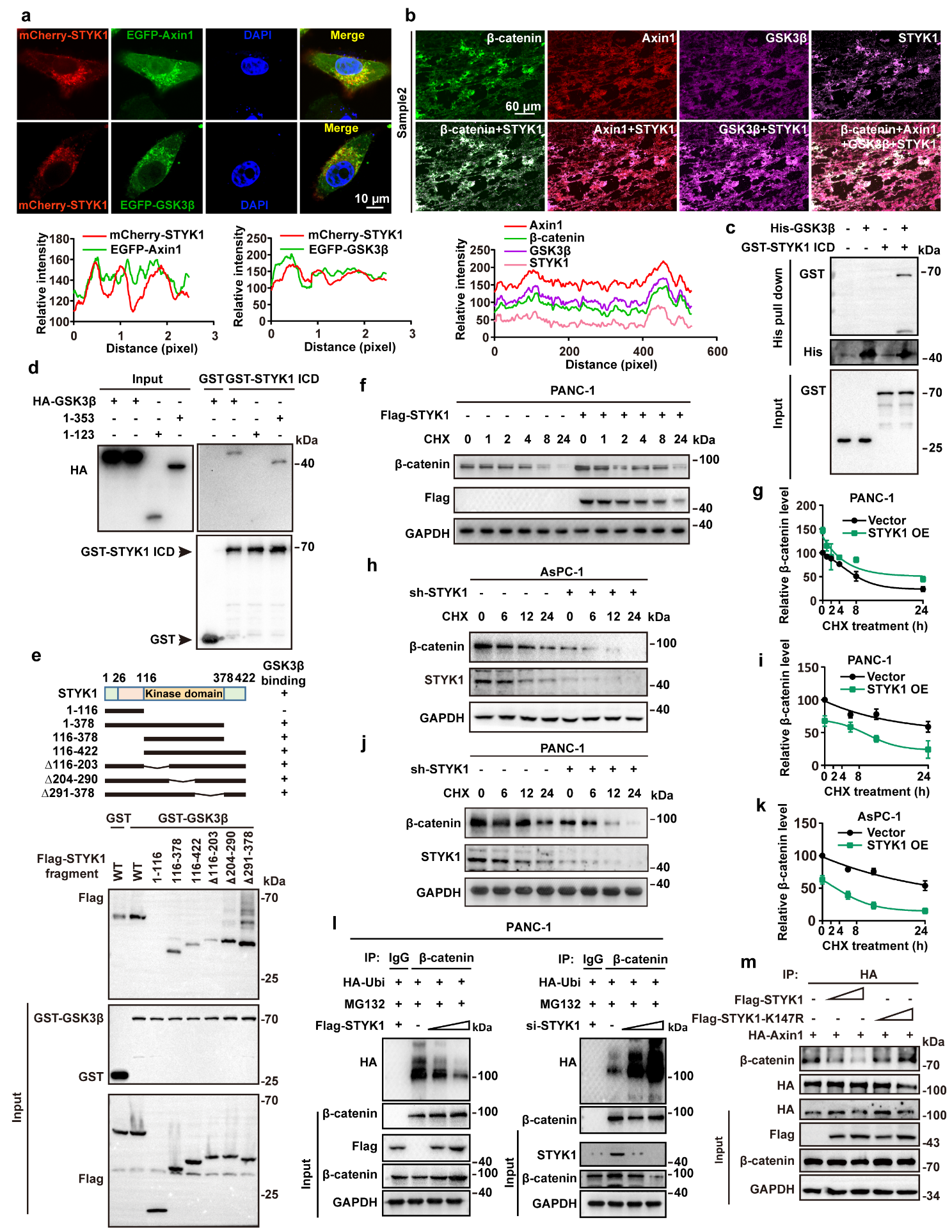
**

**Supplementary Figure 3.** STYK1 binds GSK3β and β-catenin and inhibits cytoplasmic GSK3β activity thus stabilizing β-catenin, related to Fig.3.

**a** The co-localization of mCherry-tagged STYK1 and EGFP-tagged Axin1 and GSK3β in HeLa cells was analyzed with confocal microscopy. Scale bar: 10 μm. **b** Immunofluorescent staining of endogenous STYK1, Axin1, GSK3β, and β-catenin in pancreatic cancer clinical tissues. Plot profile analysis of protein colocalization. The X-axis represents the distance in pixels along the region of interest, and the Y-axis indicates the relative fluorescence intensity of each channel. Scale bar: 60 μm. **c** Externally puriﬁed GST-tagged STYK1 were incubated with His-tagged GSK3β, the mixture was pulled down by NI-sepharose and then the precipitates were subjected to western blotting. **d, e** Indicated GSK3β and STYK1 truncation or deletion mutants obtained from transfected HEK293T cells were incubated with GST-tagged STYK1 ICD or GSK3β and then the precipitates were subjected to western blotting. The schematic diagram of GSK3β and STYK1 truncation generation and STYK1-GSK3β mutants interacting domain were shown. **f-k** Western blotting analysis of β-catenin after STYK1 depletion or overexpression in the CHX chase assay in PANC-1 or AsPC-1 cells. **l** Western blotting analysis of the level of β-catenin ubiquitination in PANC-1 cell lysates after STYK1 overexpression or depletion. **m** The interaction between exogenous HA-tagged Axin1 and β-catenin in AsPC-1 cell lysates upon STYK1 and its K147R mutant overexpression was analyzed by western blotting. Data were represented as mean ± SD, *P < 0.05; **P < 0.01; ***P < 0.001.

**
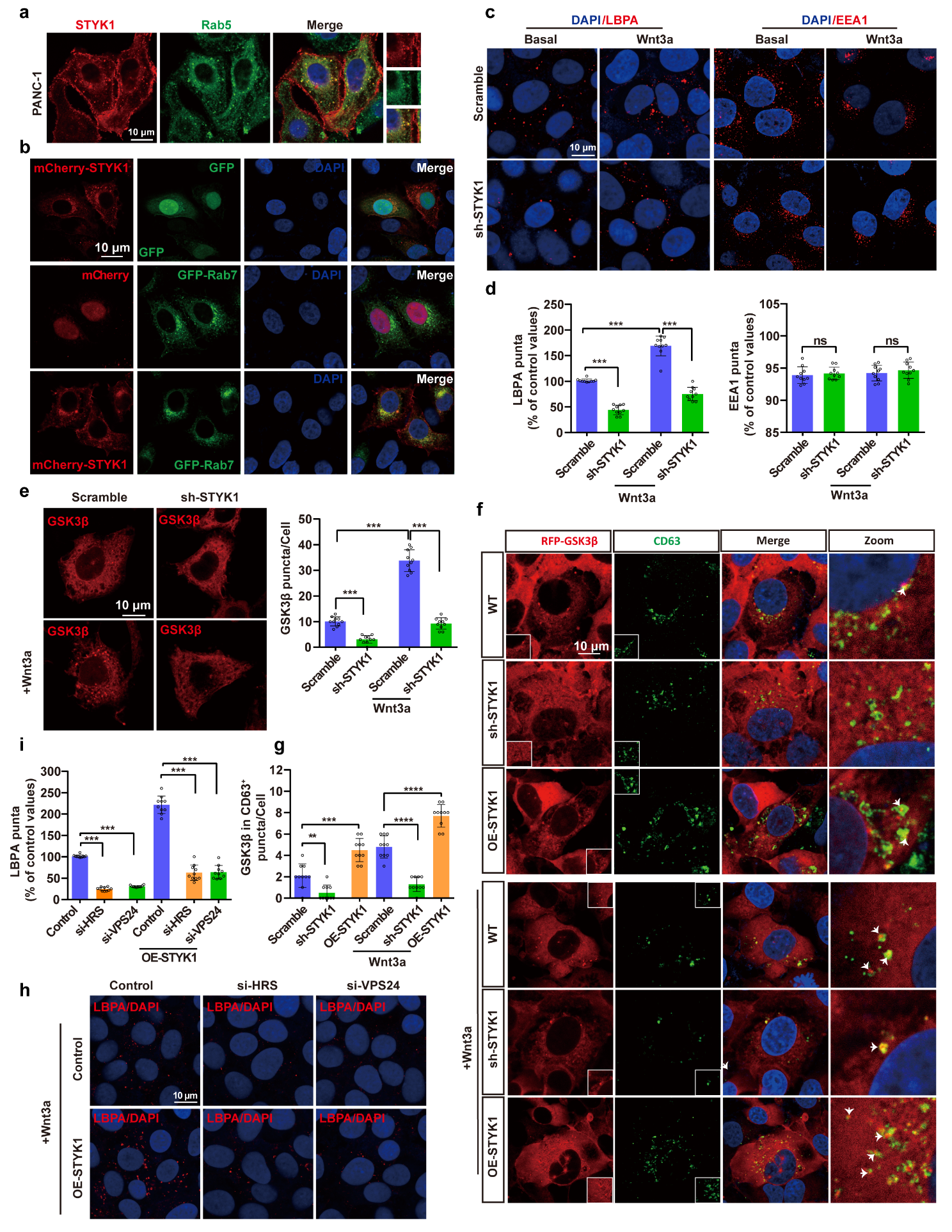
**

**Supplementary Figure 4.** STYK1 promotes cell membrane-associated GSK3β sequestration in an ESCRT-dependent manner, related to Fig.4.

**a** Confocal microscopy images of endogenous STYK1 and Rab5 in HeLa cells. Scale bar: 10 μm. **b** Confocal microscopy images of mCherry-tagged STYK1 and EGFP-tagged Rab7 in HeLa cells. Scale bar: 10 μm. **c, d** Representative immunofluorescence images and the quantification of LBPA and EEA1 puncta upon Wnt3a treatment in the PANC-1 cell lysates. Scale bar: 10 μm. **e** Representative immunofluorescence images and the quantification of GSK3β puncta upon STYK1 depletion with or without Wnt3a treatment in the PANC-1 cell lysates. Scale bar: 10 μm. **f, g** Representative immunofluorescence images and the quantification of the co-localization between CD63 and RFP-tagged GSK3β upon STYK1 depletion or expression with or without Wnt3a treatment. Scale bar: 10 μm. **h, i** Representative immunofluorescence images and the quantification of LBPA puncta upon Wnt3a treatment in the PANC-1 cell lysates with HRS or VPS24 knockdown. Scale bar: 10 μm.


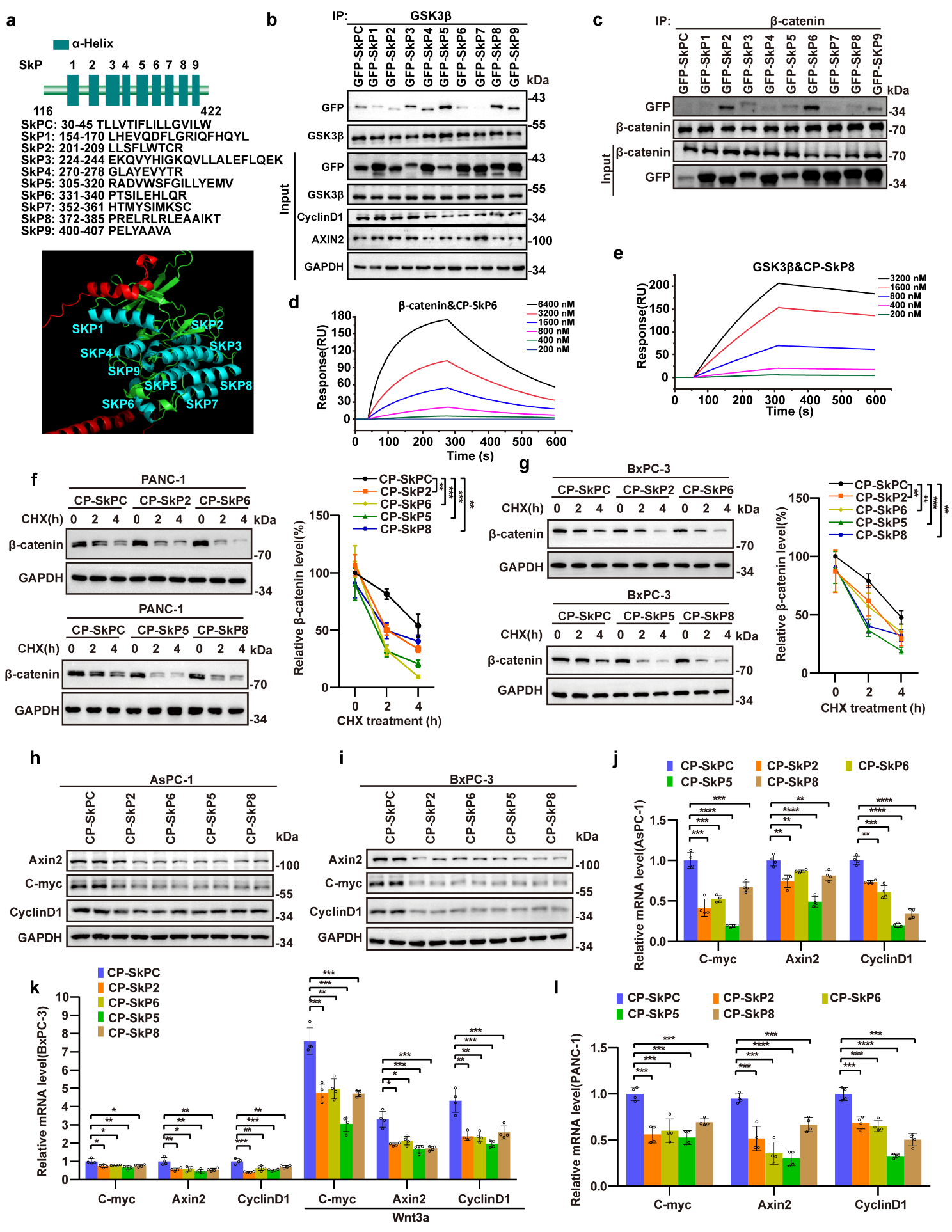


**Supplementary Figure 5.** Disrupting STYK1-β-catenin or STYK1-GSK3β interaction inhibits GSK3β sequestration and subsequent Wnt/β-catenin signaling, related to Fig.5.

**a** Predicted α-helical peptide(s) of STYK1 kinase domain (116aa-422aa) region. The secondary structure of the STYK1 kinase domain was analyzed using the predictive I-TASSER server. **b** Western blotting analysis of the binding of GSK3β and GFP-tagged STYK1-driving peptides using indicated antibodies. **c** Western blotting analysis of the binding of β-catenin and GFP-tagged STYK1-driving peptides using indicated antibodies. **d, e** Kinetic interactions of chemically synthesized cell-penetrating peptide TAT-linked peptides with β-catenin and GSK3β were determined by surface plasmon resonance analyses. **f, g** Western blotting analysis of the level of β-catenin after STYK1-driving peptides treatment in the CHX chase assay in PANC-1 and BxPC-3 cells. **h-l** mRNA and protein levels of Wnt target genes CyclinD1, C-myc and Axin2 in AsPC-1, PANC-1 and BxPC-3 cell lysates upon STYK1-driving peptides treatment. Data were represented as mean ± SD, *P < 0.05; **P < 0.01; ***P < 0.001.


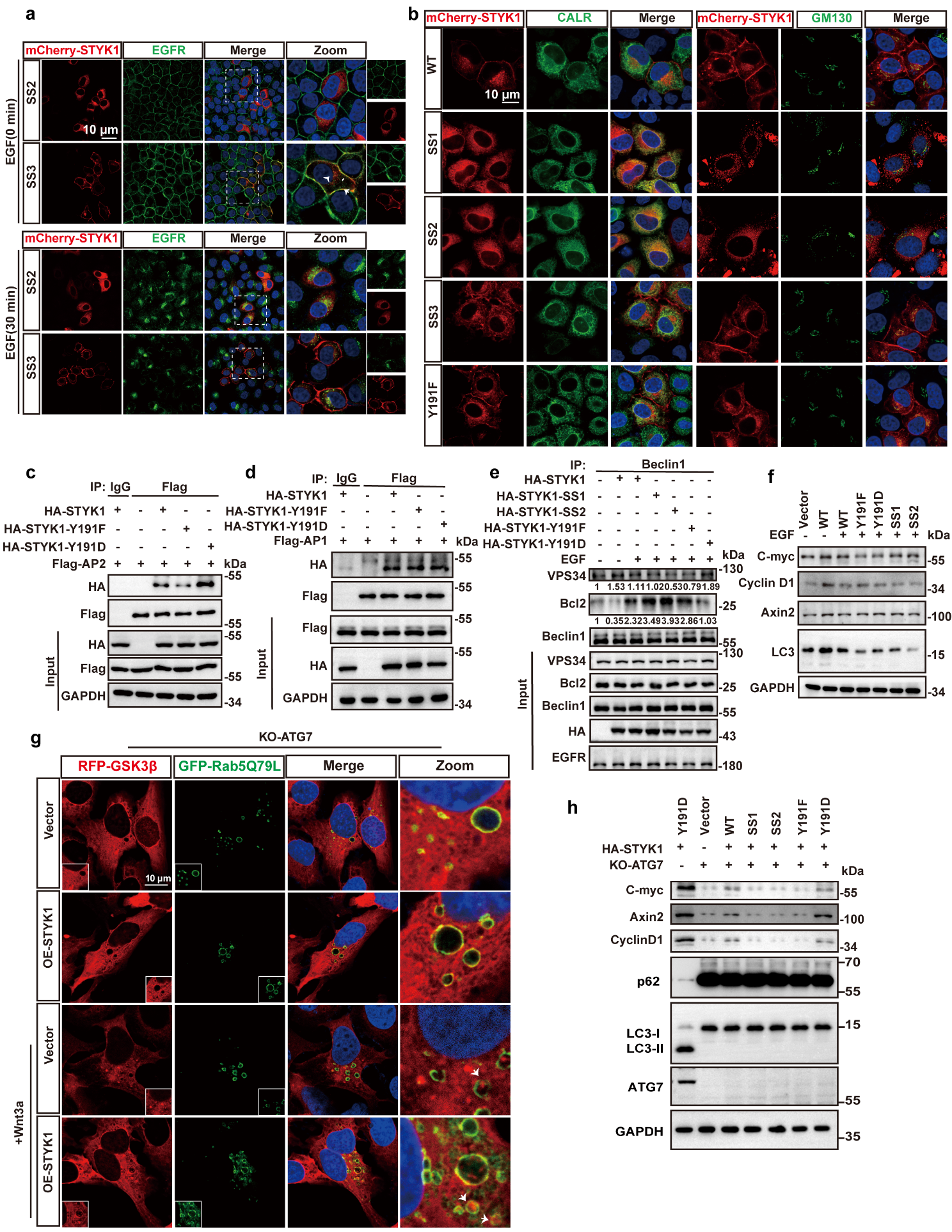


**Supplementary Figure 6.** AP2-mediated STYK1 internalization enhances GSK3β sequestration through autophagy activation, related to Fig.6.

**a** Co-localization of mCherry-tagged STYK1 mutants SS2 and SS3 with EGFR. Scale bar: 10 μm. **b** Co-localization of mCherry-tagged wild-type STYK1 and its SS1, SS2, SS3, and Y191F mutants with endoplasmic protein CALR and Golgi membranous protein GM130, respectively. Scale bar: 10 μm. **c, d** The interaction between Flag-tagged μ subunits from the AP1 and AP2 complexes and HA-tagged wild-type STYK1 and its Y191F, Y191D mutants were analyzed by co-IP using indicated antibodies. **e** The interaction of endogenous VPS34 and Bcl2 with Beclin1 in wild-type STYK1 and its SS1, SS2, SS3, Y191F, and Y191D mutants transfected PANC-1 cell lysates were analyzed by co-IP using indicated antibodies. **f** The levels of C-myc, CyclinD1, Axin2, and LC3 in wild-type STYK1 and its SS1, SS2, Y191F, and Y191D mutants transfected PANC-1 cell lysates with or without EGF treatment were analyzed. **g** Representative immunofluorescence images of RFP-tagged GSK3β and EGFP-tagged Rab5 Q79L mutant in ATG7^-/-^ U2OS cells upon STYK1 overexpression with or without Wnt3a treatment. Scale bar: 10 μm. **h** The levels of C-myc, Axin2, p62, and LC3 in ATG7 knockout U2OS cells transfected with wild-type STYK1 and its mutants SS1, SS2, Y191F and Y191D were analyzed.


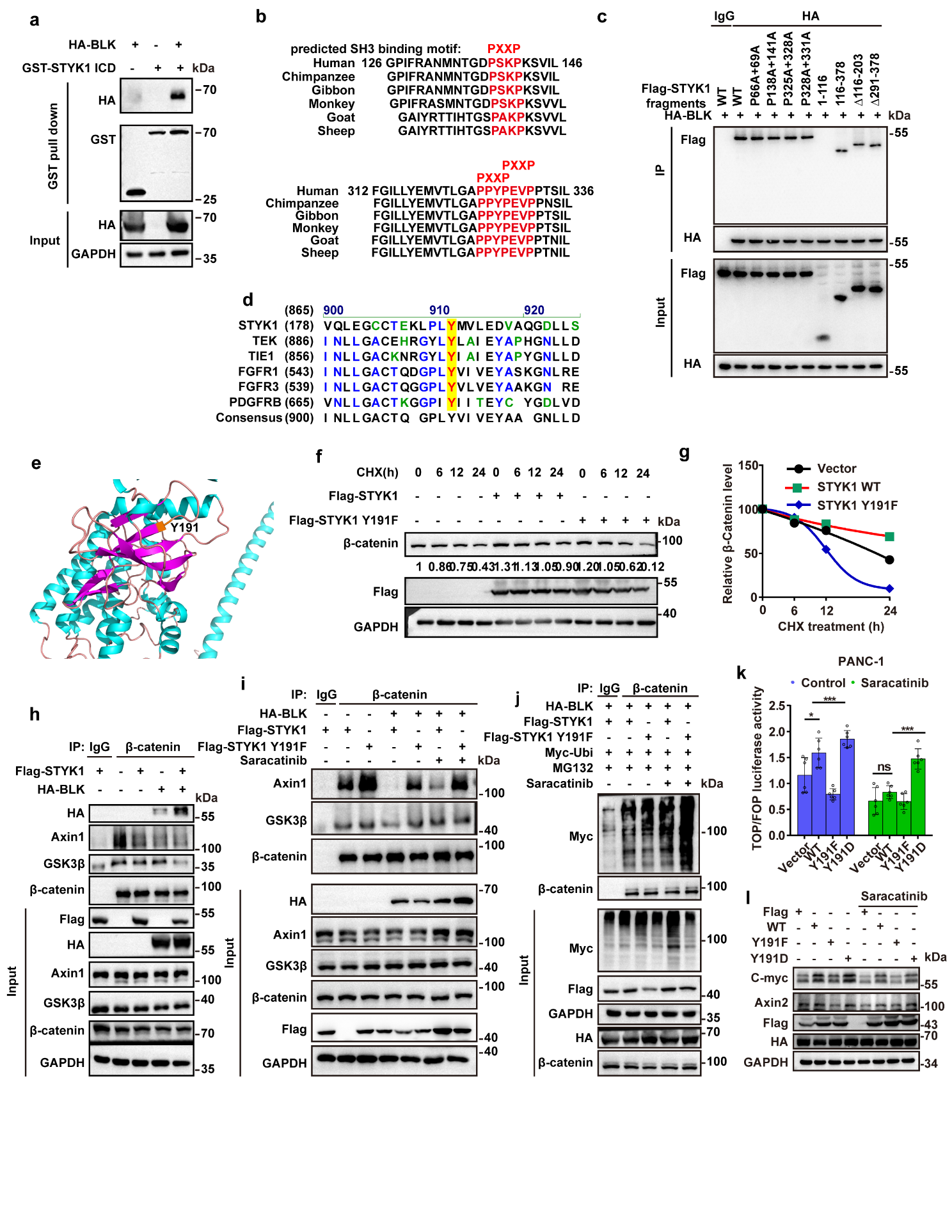


**Supplementary Figure 7.** Phosphorylation of STYK1 at Y191 by BLK kinase enhances pancreatic cancer cell proliferation, related to Fig.7.

**a** *In vitro* puriﬁed GST-STYK1 ICD were incubated with HEK293T cell lysates with or without ATP addition, and the reaction products were subjected to western blotting. **b, c** The interaction of HA-tagged BLK with wild-type STYK1 and its mutants that deleted the predicted SH3 binding “PXXP” motif by co-IP assay. **d, e** The conservation of STYK1 in the tyrosine kinase members and view of the STYK1 Y191 localization in the predicted structure of STYK1 protein. **f, g** Western blotting analysis and the quantification of β-catenin level in wild-type STYK1 or its Y191F mutant transfected PANC-1 cells in the CHX chase assay. **h** The interaction between endogenous Axin1, and GSK3β with β-catenin in PANC-1 cell lysates upon STYK1 or BLK overexpression were analyzed by western blotting. **i** The interaction between endogenous Axin1, GSK3β with β-catenin in PANC-1 cell lysates upon STYK1, STYK1 Y191F, or BLK overexpression with or without saracatinib treatment was analyzed by western blotting. **j** Western blotting analysis of the level of β-catenin ubiquitination in PANC-1 cell lysates upon STYK1, STYK1 Y191F, or BLK overexpression with or without saracatinib treatment was analyzed by western blotting. **k** Relative Wnt reporter luciferase activity (TOP/FOPflash) in PANC-1 cells upon STYK1, STYK1 Y191F, or Y191D mutants transfection with or without saracatinib treatment. **l** Protein levels of Wnt target genes C-myc and Axin2 in PANC-1 cell lysates upon STYK1, STYK1 Y191F, or Y191D mutants transfection with or without saracatinib treatment. Data were represented as mean ± SD, *P < 0.05; **P < 0.01; ***P < 0.001. Data were presented as mean ± SD.


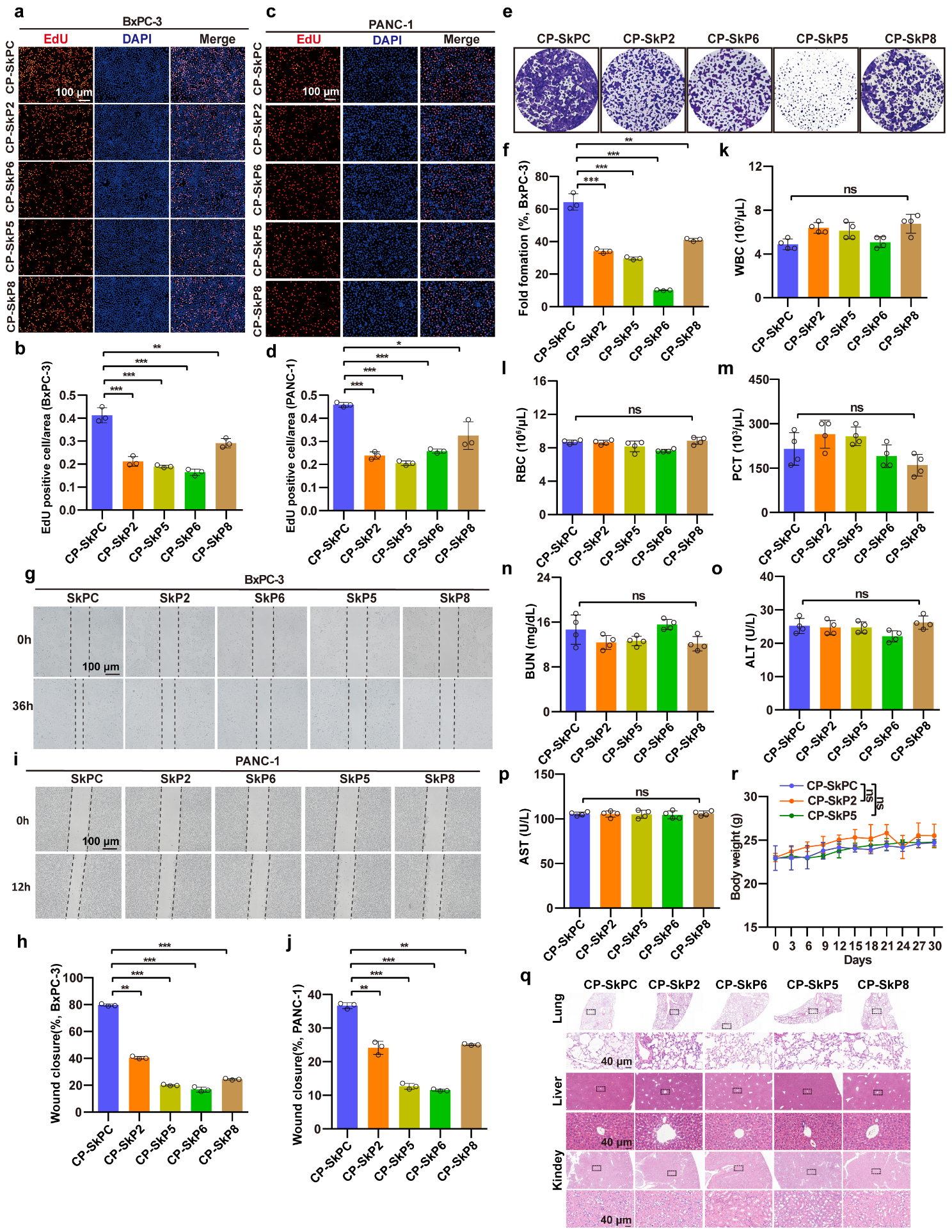


**Supplementary Figure 8.** STYK1-driving peptides efficiently inhibit pancreatic cancer development, related to Fig.8.

**a-d** Representative images and quantification of DNA synthesis in the BxPC-3 and AsPC-1 cells treated with control, CP-SkP2, CP-SkP6, CP-SkP5, and CP-SkP8 peptides. **e, f** Representative images and quantification of the colony formation assays of BxPC-3 cells treated with control, CP-SkP2, CP-SkP6, CP-SkP5, and CP-SkP8 peptides. (**G-J**) Representative images and quantification of the wound healing assays of PANC-1 and BxPC-3 cells treated with indicated peptides. **k-p** Serum concentration of BUN, ALT, AST, WBC, RBC, and PCT in mice treated with indicated peptides. **q** Representative H&E images of normal organs including lungs, livers, and kidneys were collected from mice treated with indicated peptides. **r** The quantification of the body weight in mice treated with indicated peptides.


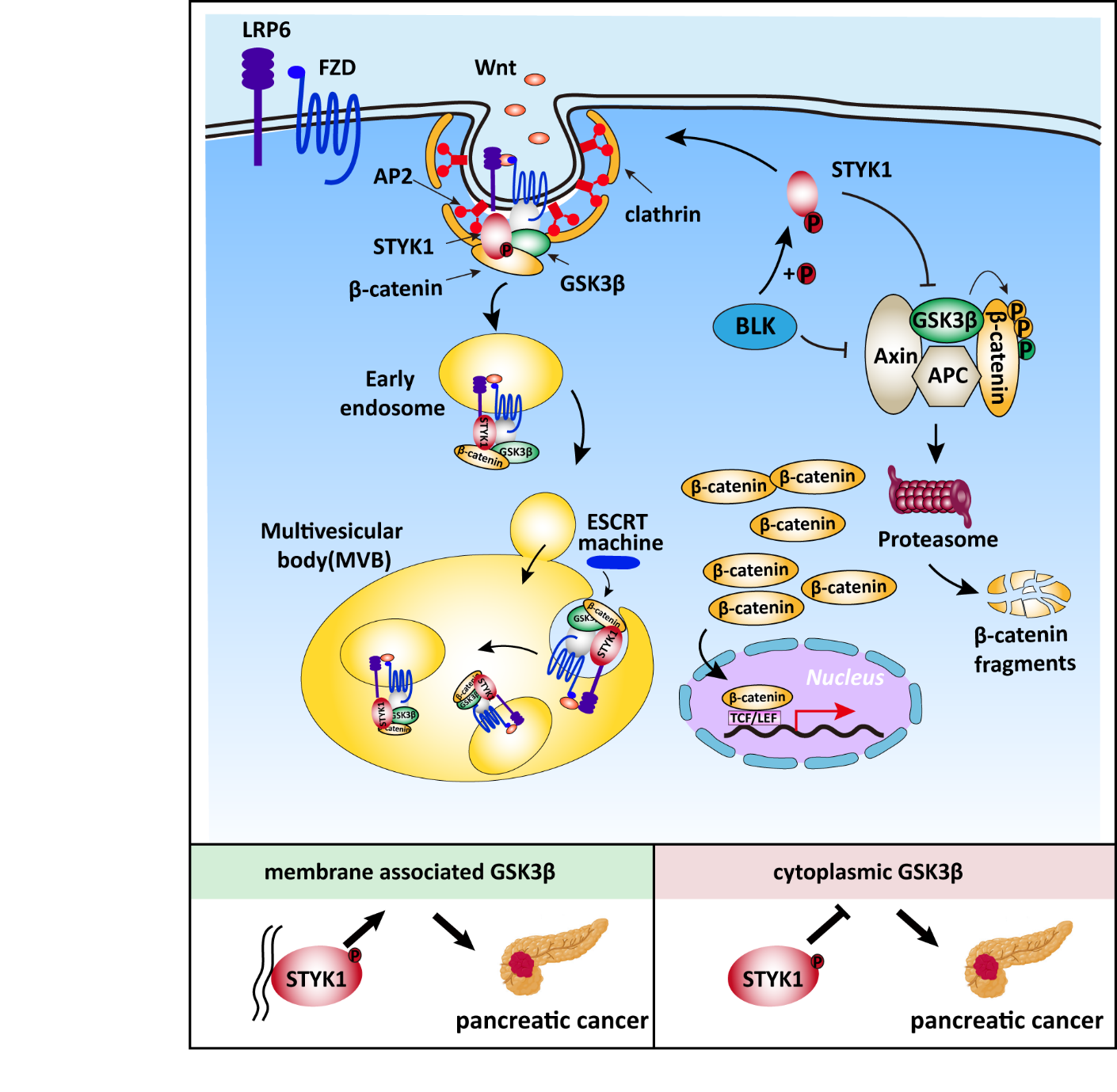


**Supplementary Figure 9.** Schematic diagram of the biological role of clathrin/AP2-mediated internalization of STYK1 and membrane-associated GSK3β sequestration in Wnt/β-catenin signal activation and pancreatic cancer development.

# References

1. Zhou C. et al. TSPAN1 promotes autophagy flux and mediates cooperation between WNT-CTNNB1 signaling and autophagy via the MIR454-FAM83A-TSPAN1 axis in pancreatic cancer. *Autophagy* **17,** 3175-3195 (2021).

2. Chen K. et al. Metformin suppresses cancer initiation and progression in genetic mouse models of pancreatic cancer. *Mol Cancer* **16,** 131 (2017).

3. Zhang R. et al. RUNDC1 inhibits autolysosome formation and survival of zebrafish via clasping ATG14-STX17-SNAP29 complex. *Cell Death Differ* **30,** 2231-2248 (2023).

4. Livak KJ, Schmittgen TD. Analysis of relative gene expression data using real-time quantitative PCR and the 2(-Delta Delta C(T)) Method. *Methods* **25,** 402-408 (2001).

5. Zhang X. et al. Phosphorylated PTTG1 switches its subcellular distribution and promotes β-catenin stabilization and subsequent transcription activity. *Oncogene* **42,** 2439-2455 (2023).
